# The DNA replication initiation protein DnaD recognises a specific strand of the *Bacillus subtilis* chromosome origin

**DOI:** 10.1093/nar/gkad277

**Published:** 2023-04-24

**Authors:** Charles Winterhalter, Simone Pelliciari, Daniel Stevens, Stepan Fenyk, Elie Marchand, Nora B Cronin, Panos Soultanas, Tiago R D Costa, Aravindan Ilangovan, Heath Murray

**Affiliations:** Centre for Bacterial Cell Biology, Biosciences Institute, Newcastle University, Newcastle Upon Tyne NE2 4AX, UK; Centre for Bacterial Cell Biology, Biosciences Institute, Newcastle University, Newcastle Upon Tyne NE2 4AX, UK; Centre for Bacterial Cell Biology, Biosciences Institute, Newcastle University, Newcastle Upon Tyne NE2 4AX, UK; Centre for Bacterial Cell Biology, Biosciences Institute, Newcastle University, Newcastle Upon Tyne NE2 4AX, UK; Centre for Bacterial Cell Biology, Biosciences Institute, Newcastle University, Newcastle Upon Tyne NE2 4AX, UK; LonCEM, London Consortium for Cryo-EM, The Francis Crick Institute, London NW1 1AT, UK; Biodiscovery Institute, School of Chemistry, University of Nottingham, Nottingham NG7 2RD, UK; Centre for Bacterial Resistance Biology, Department of Life Sciences, Imperial College London, London SW7 2AZ, UK; Department of Biochemistry, School of Biological and Behavioural Sciences, Queen Mary University of London, London, UK; Centre for Bacterial Cell Biology, Biosciences Institute, Newcastle University, Newcastle Upon Tyne NE2 4AX, UK

## Abstract

Genome replication is a fundamental biological activity shared by all organisms. Chromosomal replication proceeds bidirectionally from origins, requiring the loading of two helicases, one for each replisome. However, the molecular mechanisms underpinning helicase loading at bacterial chromosome origins (*oriC*) are unclear. Here we investigated the essential DNA replication initiation protein DnaD in the model organism *Bacillus subtilis*. A set of DnaD residues required for ssDNA binding was identified, and photo-crosslinking revealed that this ssDNA binding region interacts preferentially with one strand of *oriC*. Biochemical and genetic data support the model that DnaD recognizes a new single-stranded DNA (ssDNA) motif located in *oriC*, the *D*naD *R*ecognition *E*lement (DRE). Considered with single particle cryo-electron microscopy (cryo-EM) imaging of DnaD, we propose that the location of the DRE within *oriC* orchestrates strand-specific recruitment of helicase during DNA replication initiation. These findings significantly advance our mechanistic understanding of bidirectional replication from a bacterial chromosome origin.

## Graphical Abstract

**Graphical Abstract:**
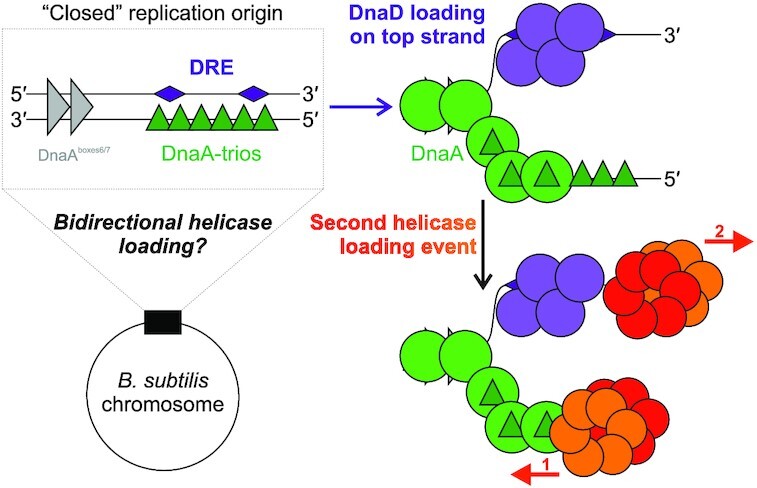
In *Bacillus subtilis*, the basal unwinding sequence at the origin of replication *oriC* was known to comprise two DnaA boxes (DnaA^boxes6/7^, grey triangles) and a single-strand DNA binding region on the bottom strand (DnaA-trios, green triangles). The master initiator protein DnaA (green circles) binds both of these regions and can provide a scaffold to load a first helicase on bottom strand. Here we discovered a new element of the unwinding region located opposite the DnaA-trios and recognised by the replication protein DnaD, which we named the *D*naD *R*ecognition *E*lement (DRE). We propose that binding of DnaD to the DRE promotes a second helicase loading event, providing a pathway to achieve bidirectional DNA replication initiation at a bacterial chromosome origin.

## INTRODUCTION

Faithful chromosome replication is universally essential to sustain life and initiates from specific loci called origins. Although the mechanisms triggering the onset of DNA replication are distinct across the phyla of life, all species require the loading of unwinding machines termed helicases prior to duplication of the genetic material (1). In bacteria, replication initiation generally occurs from a single origin (*oriC*) and proceeds bidirectionally towards an endpoint region called the terminus, located approximately equidistant from the origin on the circular chromosome (2–5). To initiate this process, two toroid hexameric helicases need to be loaded at the origin, one around each strand (6). Despite fifty years of evidence for bidirectional DNA replication initiation in bacteria (7–9), the molecular mechanism for dual helicase loading at *oriC* remains unclear (10).

**Figure 1.**
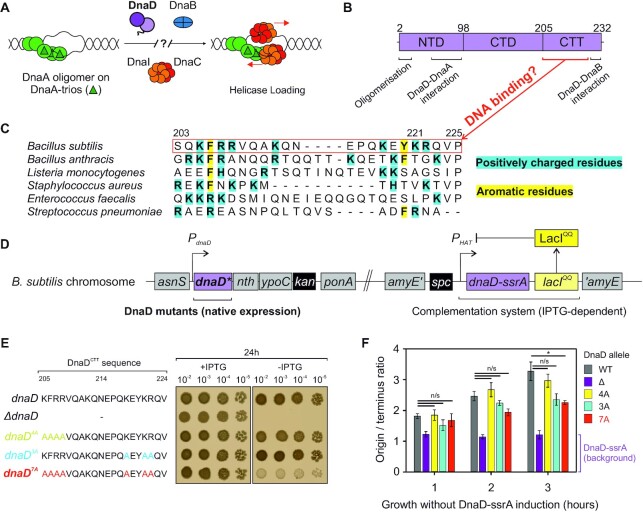
Identification of important DNA binding residues in *B. subtilis* DnaD. (**A**) Schematics of the helicase loading pathway in *B. subtilis* showing sequential recruitment of DnaA, DnaD, DnaB and the helicase complex DnaI-DnaC. (**B**) DnaD primary structure mapped with regions that are important for oligomerisation and interactions with DnaA and DnaB. NTD denote the N-Terminal Domain, CTD the C-Terminal Domain and CTT the C-Terminal Tail of DnaD. Amino acid sequence information shown in panel (**C**) suggested a potential DNA binding interface. (C) Protein sequence alignment of DnaD homologs showing the recurrence of positively charged (cyan) and aromatic residues (yellow) within the DnaD^CTT^. (**D**) Schematics of the complementation system used to screen the function of DnaD mutants in *B. subtilis*. Mutants are integrated at the endogenous *dnaD* locus and an inducible ectopic copy of *dnaD* (*dnaD-ssrA*) allows functional complementation by the addition of IPTG to the culture medium. The function of DnaD mutants can be determined upon removal of IPTG leading to repression of the *dnaD-ssrA* copy and degradation of DnaD-ssrA. (**E**) Spot titre analysis showing that multiple DnaD^CTT^ substitutions targeting residues highlighted in panel (C) were required to produce a growth phenotype as observed in *dnaD^7A^*. The presence or absence of IPTG indicates the induction state of the ectopic *dnaD-ssrA* cassette. *dnaD* (CW162), *ΔdnaD* (CW197), *dnaD^4A^* (CW412), *dnaD^3A^* (CW415), *dnaD^7A^* (CW647). (**F**) Marker frequency analysis of DnaD^CTT^ variants indicates that cells harbouring the 7A mutation significantly under-initiate DNA replication as measured by quantitative PCR. * shows a statistically significant difference (*P*-value of 0.029) and n/s indicates non-significant differences. The residual DNA replication initiation rate following DnaD-ssrA depletion in the absence of *dnaD* is shown in purple. Error bars show the standard error of the mean for three biological replicates. WT (CW162), Δ (CW197), 4A (CW412), 3A (CW415) and 7A (CW647).

In *Bacillus subtilis*, DNA replication is initiated by five proteins that are sequentially localised to the chromosome origin (Figure 1A): DnaA, DnaD, DnaB and the DnaC:DnaI complex (helicase and AAA+ (*A*TPase *A*ssociated with various cellular *A*ctivities) loader, respectively) (11). The ubiquitous bacterial initiator DnaA binds at *oriC* and promotes open complex formation to allow helicase loading. DnaA is recruited to the origin by a conserved helix-turn-helix motif in domain IV, mediating binding to asymmetric double-strand DNA (dsDNA) sequences called DnaA-boxes (consensus 5′-TTATCCACA-3′) (12–14). This promotes formation of a DnaA^ATP^ oligomer through extensive contacts between the protein's AAA+ motifs, allowing it to engage a single strand of the DNA duplex (ssDNA)(15) at specific trinucleotide motifs called DnaA-trios (consensus 3′-GAT-5′) (16–18).

A direct interaction between the AAA+ motifs of DnaA and of a helicase loader protein (*Aquifex aeolicus* DnaC) have been proposed to orchestrate loading of one helicase onto the strand bound by the DnaA oligomer (Figure 1A) (19). However, the mechanism for loading a helicase onto the opposite strand is unclear. Recently, a region of DnaA domain I was identified to be essential for the recruitment of DnaD to *oriC* (20), raising the possibility that this interaction might guide the deposition of a second helicase at the replication origin.

The DnaD protein is comprised of an N-terminal domain (DnaD^NTD^), a C-terminal domain (DnaD^CTD^) and a C-terminal tail (DnaD^CTT^). A recent alanine scan characterised the residues of DnaD necessary for its functions *in vivo*, identifying those required for tetramerization and protein:protein interactions with DnaA and DnaB (Figure 1B) (20). Conspicuously however, this analysis did not identify any residues required for DNA binding. Previous studies indicated that DnaD displays a general affinity for DNA (21–26), with the ability to untwist dsDNA at high protein concentrations (24,25). Furthermore, analysis of DnaD truncations *in vitro* strongly suggested that the DNA binding activity of DnaD was located in the C-terminal tail (21–23). Nonetheless, because specific mutations inactivating the DNA binding activity of DnaD have not been identified, the physiological relevance of DnaD binding to DNA was uncertain.

Here we identify DnaD variants specifically defective for binding ssDNA. Characterization of these DnaD proteins indicates that DnaD recognises a specific strand of *oriC* via a new ssDNA binding motif, the *D*naD *R*ecognition *E*lement (DRE). The location of the DRE opposite the DnaA-trios suggests a mechanism for directing strand-specific helicase loading during DNA replication initiation at the *B. subtilis* chromosome origin.

## MATERIALS AND METHODS

### Reagents

Nutrient agar (NA; Oxoid) was used for routine selection and maintenance of both *B. subtilis* and *E. coli* strains. Supplements were added as required: ampicillin (100 μg/ml), chloramphenicol (5 μg/ml), kanamycin (5 μg/ml), spectinomycin (50 μg/ml in *B. subtilis*, 100 μg/ml in *E. coli*), tetracycline (10 μg/ml), erythromycin (1 μg/ml) in conjunction with lincomycin (25 μg/ml), X-gal (0.01% v/v), IPTG (0.1 mM unless indicated otherwise). All chemicals and reagents were obtained from Sigma-Aldrich unless otherwise noted. Antibodies were purchased from Eurogentec. Plasmid extractions were performed using Qiagen miniprep kits. Other reagents used for specific techniques are listed within the method details.

### Biological resources: *B. subtilis* strains

*B. subtilis* strains are listed in Supplementary Table S1 and were propagated at 37°C in Luria-Bertani (LB) medium unless stated otherwise in method details. Transformation of competent *B. subtilis* cells was performed using an optimized two-step starvation procedure as previously described (27,28). Briefly, recipient strains were grown overnight at 37°C in transformation medium (Spizizen salts supplemented with 1 μg/ml Fe-NH_4_-citrate, 6 mM MgSO_4_, 0.5% w/v glucose, 0.02 mg/ml tryptophan and 0.02% w/v casein hydrolysate) supplemented with IPTG where required. Overnight cultures were diluted 1:17 into fresh transformation medium supplemented with IPTG where required and grown at 37°C for 3 hours with continual shaking. An equal volume of prewarmed starvation medium (Spizizen salts supplemented with 6 mM MgSO_4_ and 0.5% w/v glucose) was added and the culture was incubated at 37°C for 2 h with continual shaking. DNA was added to 350 μl cells and the mixture was incubated at 37°C for 1 h with continual shaking. 20–200 μl of each transformation was plated onto selective media supplemented with IPTG where required and incubated at 37°C for 24–48 h. The genotype of all chromosomal origin and *dnaD* mutants was confirmed by DNA sequencing. Descriptions, where necessary, are provided below.

CW483 [*trpC2 Δ6*] was generated via CrispR-Cas genome editing to remove the chloramphenicol cassette from HM1774 [*trpC2 Δ6(pks::cat)*] using an optimised procedure as previously described (29).

### Biological resources: *E*.*coli* strains and plasmids

*E. coli* transformations were performed in CW198 via heat-shock following the Hanahan method (30) for plasmids harbouring *dnaD* or *lexA* and propagated in LB with appropriate antibiotics at 37°C unless indicated otherwise in method details. Plasmids are listed in the Supplementary Table S2 (sequences are available upon request). DH5α [F^−^ Φ80*lac*ZΔM15 Δ(*lac*ZYA-*arg*F) U169 *rec*A1 *end*A1 *hsd*R17(r_k_^−^, m_k_^+^) *pho*A *sup*E44 *thi*-1 *gyr*A96 *rel*A1 λ^−^] (31) was used for other plasmids construction. Descriptions, where necessary, are provided below.

pCW270, pCW365, pCW366, pCW464 and pCW495 were generated by ligase-free cloning via two-step assembly processes using oligonucleotides listed in Supplementary Table S3. FastCloning (32) was used with minor modifications. PCR products (15 μl from a 50 μl reaction) were mixed and then subjected to a heating/cooling regime: two cycles at 98°C for 2 min then 25°C for 2 min, followed by one cycle at 98°C for 2 min and a final cycle at 25°C for 60 min. After cooling, *DpnI* restriction enzyme (1 μl) was added to digest parental plasmids and the mixtures were incubated at 37°C for ∼4 h. Following digestion 10 μl of the PCR mixture was transformed into chemically competent *E. coli*. Where several primer pairs are listed for the construction of a single plasmid (Multi-step assembly column in Supplementary Table S3), multiple rounds of ligase free cloning were performed to obtain the final constructs.

pCW304, pCW305, pCW310, pCW313, pCW339, pCW364, pCW406 and pCW407 were generated by Quickchange mutagenesis using the oligonucleotides listed in Supplementary Table S3.

pCW568 was linearly assembled using Gibson assembly in a two-step *in vitro* process using the oligonucleotides listed in Supplementary Table S3. First, *B. subtilis* 168CA genomic DNA and pCW464 were used as templates to integrate a tetracycline resistance cassette downstream of the *lexA* coding sequence and this assembly was PCR-amplified. Second, resulting PCR products and pCW464 were used as templates to replace *lexA* by *mNeonGreen-lexA* and this assembly was PCR-amplified to transform CW483.

### Biological resources: oligonucleotides

Desthiobiotin-labelled oligonucleotides were purchased from IDT. All other oligonucleotides were purchased from Eurogentec. Oligonucleotides used for plasmid construction and for qPCR are listed in Supplementary Table S3.

### Statistical analyses

Statistical analysis was performed using Student's *t*-tests and *P*-values are given in figure legends. The exact value of *n* is given in method details and represents the number of biological repeats for an experiment. Tests were based on the mean of individual biological replicates and error bars indicate the standard error of the mean (SEM) across these measurements. Differences were considered as significant if their associated *P*-value was <0.05. For ChIP experiments showing DnaA and DnaD depletion from the origin, the standard error of the mean was propagated by addition of the error from individual terms.

### Microscopy

To visualize cells by microscopy during the exponential growth phase, starter cultures were grown in imaging medium (Spizizen minimal medium supplemented with 0.001 mg/mL ferric ammonium citrate, 6 mM magnesium sulphate, 0.1 mM calcium chloride, 0.13 mM manganese sulphate, 0.1% w/v glutamate, 0.02 mg/ml tryptophan) with 0.5% v/v glycerol, 0.2% w/v casein hydrolysate and 0.1 mM IPTG at 37°C. Saturated cultures were diluted 1:100 into fresh imaging medium supplemented with 0.5% v/v glycerol and 0.1 mM IPTG and allowed to grow for three mass doublings. For DnaD mutants, early log cells were then spun down for 5 min at 9000 rpm, resuspended in the same medium lacking IPTG and further incubated for up to 3 h before imaging. For origin mutants, cells were grown in PAB at 20°C up to mid-exponential phase and 200 μl cells were further incubated with DAPI (1 μg/ml) for 15 min. Bacterial membranes were imaged by adding Nile red (1 μg/ml) directly to the agarose pad.

Cells were mounted on ∼1.4% agar pads (in sterile ultrapure water) and a 0.13- to 0.17-mm glass coverslip (VWR) was placed on top. Microscopy was performed on an inverted epifluorescence microscope (Nikon Ti) fitted with a Plan Apochromat Objective (Nikon DM 100×/1.40 Oil Ph3). Light was transmitted from a CoolLED pE-300 lamp through a liquid light guide (Sutter Instruments), and images were collected using a Prime CMOS camera (Photometrics). The fluorescence filter sets were from Chroma: GFP (49002, EX470/40 (EM), DM495lpxr (BS), EM525/50 (EM)), mCherry (49008, EX560/40 (EM), DM585lprx (BS), EM630/75 (EM)) and DAPI (49000, EX350/50, DM400lp, EM460/50). Digital images were acquired using METAMORPH software (version 7.7) and analysed using Fiji software (33). All experiments were independently performed at least twice, and representative data are shown.

For GFP-DnaN analyses, the number of fluorescent foci per cell was quantified using the Trackmate plugin within the Fiji software (34). Background was subtracted from fluorescence images set to detect 8–10 pixel blob diameter foci over an intensity threshold of 150 relative fluorescence units. A mask containing the detected foci was created and merged with the nucleoid (DAPI) and membrane stain (Nile red) channels, and the number of fluorescent foci per nucleoid and per cell was determined and averaged for a minimum of 100 cells from each strain that was examined. This analysis was performed for at least two independent biological repeats.

For mNeonGreen-LexA analyses, phase-contrast images were segmented using thresholding to obtain binary masks and edge detection was applied to generate cell outlines. Background was subtracted from fluorescence images and cell outlines merged with the green fluorescence channel to highlight cells with low fluorescence. Intracellular fluorescence intensity was normalised per cell area and over 100 individual cells were analysed per condition for at least two independent biological repeats.

### Phenotype analysis of *dnaD* mutants using the inducible *dnaD-ssrA* strain

Strains were grown for 18 h at 37°C on NA plates unless otherwise stated (spot-titre assays) or in Penassay Broth (PAB, plate reader experiments) either with or without IPTG (0.1 mM unless otherwise stated). All experiments were independently performed at least twice and representative data are shown.

### Immunoblot analysis

Proteins were separated by electrophoresis using a NuPAGE 4–12% Bis-Tris gradient gel run in MES buffer (Life Technologies) and transferred to a Hybond-P PVDF membrane (GE Healthcare) using a semi-dry apparatus (Bio-rad Trans-Blot Turbo). DnaA, DnaD and FtsZ were probed with affinity purified polyclonal antibodies (Eurogentec) and then detected with an anti-rabbit horseradish peroxidase-linked secondary antibody (A6154, Sigma) using an ImageQuant LAS 4000 mini digital imaging system (GE Healthcare). Detection of DnaA, DnaD and FtsZ was within a linear range. Experiments were independently performed at least twice and representative data are shown.

### Sample preparation for marker frequency analysis

Strains were grown in PAB overnight at 37°C (supplemented with IPTG for DnaD^CTT^ variants analysis). For DnaD^CTT^ variants analyses, cultures were diluted 1:40 in the morning in PAB without IPTG and ongoing cultures were adjusted to an *A*_600_ of 0.1 after harvesting samples for each timepoint throughout the course of the experiment. For origin variant analyses, overnight cultures were diluted 1:100 in PAB in the morning and cells were allowed to grow until they reached an *A*_600_ of 0.4. Five hundred microliter samples were harvested and immediately mixed with sodium azide (1% w/v final) to arrest growth and genome replication. Cultures were collected by centrifugation, the supernatant was discarded and pellets were flash frozen in liquid nitrogen before gDNA extraction via the DNeasy blood and tissue kit (Qiagen).

### Quantitative PCR (qPCR)

qPCR was performed using the Luna qPCR mix (NEB) to measure the relative amount of origin DNA compared to the terminus (marker frequency analysis), and to measure the amount of genomic loci bound to DnaA and DnaD. All PCR reactions were run in a Rotor-Gene Q Instrument (Qiagen) using individual volumes of 20 μl in a Rotor-Disc 100 (Qiagen). Standard curves were performed using Rotor-Gene Q Software v2.0.2 (Qiagen) to calculate the efficiency of each PCR reaction, which varied ∼5% between primer pairs. Following qPCR reactions, a melt curve analysis was performed to confirm the specificity of each product. Oligonucleotide primers were designed to amplify *incC* (qSF19/qSF20) and the terminus (qPCR57/qPCR58), were typically 20–25 bases in length and amplified a ∼100 bp PCR product (Supplementary Table S3).

For marker frequency analysis, serial dilutions of sample DNA and spore DNA were used as control. Individual *ori:Ter* ratios were obtained in three steps: first, every Ct value was normalised to 1/2^Ct^, the dilution factor used during the qPCR and technical triplicates were averaged to a single enrichment value; second, origin enrichment was normalised by corresponding terminus values; third, *ori:Ter* values were normalised by the enrichment obtained for spore DNA. Error bars indicate the standard error of the mean for 2–4 biological replicates.

For ChIP, serial dilutions of the immunoprecipitate and total DNA control were used as template. First, every Ct value was normalised to 1/2^Ct^, the dilution factor used during the qPCR, and technical triplicates were averaged to a single value. Second, for each primer pair the relative % IP was obtained by normalising to the amount of total DNA in the sample. Fold-change enrichment at *incC* was calculated as the ratio between the % IP at the origin compared to that of the terminus. Error bars indicate the standard error of the mean for three biological replicates.

### Protein structure representations

Protein representations were generated using the Pymol Molecular Graphics 2.1 software (35), except for cryo-EM data and its associated model fitting that were performed in Chimera (36).

### Protein purification

Wild-type *dnaA* and *dnaD* were amplified by PCR using genomic DNA from *B. subtilis* 168CA and respectively cloned into pSF14 and pSF17 containing a His^14^-SUMO tag. DnaD mutants were created from pSF17 via quickchange reactions to introduce single or multiple substitutions. Wild-type DnaD expression was optimised by using a modified TIR sequence (pSP156) (37). Plasmids were propagated in *E. coli* DH5α and transformed in BL21(DE3)-pLysS for expression. Strains were grown in LB medium at 37°C. Overnight cultures were diluted 1:100 the next morning and at *A*_600_ of 0.6, 1 mM IPTG was added before further incubation at 30°C for 4 hours. Cells were harvested by centrifugation at 7000 g for 20 min, DnaA expression pellets resuspended in 40 ml of DnaA Ni^2+^ Binding Buffer (30 mM HEPES [pH 7.6], 250 mM potassium glutamate, 10 mM magnesium acetate, 30 mM imidazole), DnaD pellets in 40 ml of DnaD Ni^2+^ Binding Buffer (40 mM Tris–HCl [pH 8.0], 0.5 M NaCl, 5% v/v glycerol, 1 mM EDTA, 20 mM imidazole), each containing 1 EDTA-free protease inhibitor tablet (Roche #37378900) and then flash frozen in liquid nitrogen. Cell pellet suspensions were thawed and incubated with 0.5 mg/ml lysozyme on ice for 1h before disruption by sonication (1 hour at 20 W with 20 s pulses/rests intervals). Cell debris were removed from the lysate by centrifugation at 24 000 g for 30 min at 4°C, then passed through a 0.2 μm filter for further clarification. Further purification steps were performed at 4°C using a FPLC with a flow rate of 1 ml/min.

Clarified lysates were applied to a 1 ml HisTrap HP column (Cytiva). For DnaA, an additional wash with 10 ml Ni^2+^ High Salt Wash Buffer (30 mM HEPES [pH 7.6], 1 M potassium glutamate, 10 mM magnesium acetate, 30 mM imidazole) was performed. Materials bound to the column were washed with 10 ml of 10% Ni^2+^ Elution Buffer (DnaA: 30 mM HEPES [pH 7.6], 250 mM potassium glutamate, 10 mM magnesium acetate, 1 M imidazole; DnaD: 40 mM Tris–HCl [pH 8.0], 0.5 M NaCl, 1 mM EDTA, 0.5 M imidazole) and proteins were eluted with a 10 ml linear gradient (10–100%) of Ni^2+^ Elution Buffer. For DnaA, fractions containing the protein were applied to a 1 ml HiTrap Heparin HP affinity column (Cytiva) equilibrated in H Binding Buffer (30 mM HEPES [pH 7.6], 100 mM potassium glutamate, 10 mM magnesium acetate) and elution was carried out with a 20 ml linear gradient (20–100%) of H Elution Buffer (30 mM HEPES [pH 7.6], 1 M potassium glutamate, 10 mM magnesium acetate). Fractions containing proteins of interest were pooled and digested with 10 μl of 10 mg/ml His^14^-Tev-SUMO protease (38). For DnaD, digestion was performed at room temperature over the course of 48 h and the same amount of His^14^-Tev-SUMO protease was added after 24 h digestion.

Digestion reactions were applied to a 1 ml HisTrap HP column to capture non-cleaved monomers, His^14^-SUMO tag and His^14^-TEV-SUMO protease. Cleaved proteins were collected in the flow-through and their purity was confirmed using SDS-PAGE. Glycerol was added (DnaA: 20% v/v final; DnaD: 10% v/v final) and proteins aliquots were flash frozen in liquid nitrogen before being stored at –80°C.

### Bpa protein labelling

Plasmids encompassing the non-canonical amino acid residue *p*-benzoyl-l-phenyl alanine (Bpa, Alfa Aesar #52083) were generated by inserting a *TAG* stop codon at the desired crosslinking position in the appropriate plasmid (construction detailed above). Electrocompetent *E. coli* BL21(DE3)-pLysS cells were co-transformed with 200 ng of the Bpa-modified expression plasmid and pSup-BpaRS-6TRN(39), which encodes the necessary tRNA and tRNA synthase to recognize the *TAG* codon and to insert Bpa at this position. Co-transformed cells were grown at 37°C overnight on plates containing the appropriate antibiotics, and colonies were harvested the next day by directly scrapping them from the plate. Bpa was dissolved in 1 M NaOH to obtain a 100 mM stock solution and then added to 2x YT media at 1 mM final concentration. After adjusting the pH of the solution to 7.0, cells collected from the transformation plate were inoculated at an optical density of 0.3, grown for 1 h at 37°C and transferred to 30°C prior to addition of 1 mM IPTG for protein expression. After 4 h of vigorous shaking at 30°C, cultures were spun down, resuspended in the appropriate Ni^2+^ Binding Buffer (40 mM Tris–HCl [pH 8.0], 0.5 M NaCl, 5% v/v glycerol, 1 mM EDTA, 20 mM imidazole and one EDTA-free protease inhibitor tablet (Roche #37378900)), flash frozen in liquid nitrogen and purified as described above.

### Bpa crosslinking

DnaD protein variants containing Bpa residues were adjusted to a final concentration of 0.1 μM and incubated with 30 nM of DNA probes in 1x strand separation buffer (10 mM HEPES–KOH (pH 7.6), 100 mM potassium glutamate, 5 mM magnesium acetate) for 10 min at room temperature. Reaction tubes were then transferred on ice and exposed to 365 nm wavelength light in a UV oven for 1 min (∼400 mJ, Boekel #234100). A loading dye (4× SDS-page, Merck) was added to the samples to a final concentration of 1x and reactions were terminated at 98°C for 5 min. Samples were loaded on a 4–12% Bis–Tris polyacrylamide gel, run in MES buffer for 45 min at 200 V and the gel was imaged using a Typhoon 9500 (GE healthcare, 600 V power).

### SEC-MALS

Experiments were conducted on a system comprising a Wyatt HELEOS-II multi-angle light scattering detector and a Wyatt rEX refractive index detector linked to a Shimadzu HPLC system (SPD-20A UV detector, LC20-AD isocratic pump system, DGU-20A3 degasser and SIL-20A autosampler) and the assays performed at 20°C. Solvent was 0.2 μm filtered before use and a further 0.1 μm filter was present in the flow path. The column was equilibrated with at least 2 column volumes of 40 mM Tris–HCl [pH 8], 500 mM NaCl, 1 mM EDTA, 20 mM imidazole, 2.5% v/v glycerol before use and flow was continued at the working flow rate until baselines for UV, light scattering and refractive index detectors were all stable.

The sample injection volume was of 100 μl, the Shimadzu LabSolutions software was used to control the HPLC and the Astra 7 software for the HELEOS-II and rEX detectors. The Astra data collection was 1 minute shorter than the LC solutions run to maintain synchronisation. Blank buffer injections were used as appropriate to check for carry-over between sample runs. Data were analysed using the Astra 7 software. Molecular weights were estimated using the Zimm fit method with degree 1 and a value of 0.179 was used for protein refractive index increment (d*n*/d*c*).

### Size-exclusion chromatography

SEC was performed with a Superdex 200 Increase 10/300GL column (Cytiva) at 4°C using a FPLC with a flow rate of 0.75 ml/min. The column was washed with two column volumes of MilliQ water before equilibration with 2.2 column volumes of DnaD buffer without glycerol (40 mM Tris–HCl [pH 8], 500 mM NaCl, 1 mM EDTA, 20 mM imidazole). Proteins were spun down at 17 000 g for 2 min to remove aggregates and 500 μl samples were applied to the column. A total of 1.5 column volumes of DnaD buffer without glycerol was passed through the column and data was extracted via the Unicorn 7 software.

### Sequence alignments

Multiple protein sequence alignments were performed using the Clustal Omega tool (40). DNA sequences were aligned via MUSCLE (41).

### Chromatin immunoprecipitation (ChIP)

Chromatin immunoprecipitation was performed as previously described (42) with minor modifications. Strains were grown overnight at 30°C in Spizizen salts supplemented with tryptophan (20 μg/ml), glutamate (0.1% w/v), glucose (0.5% w/v) and casamino acid (0.2% w/v). The following day cultures were diluted 1:100 into fresh medium and allowed to grow to an *A*_600_ of 0.4. Samples were resuspended in PBS and cross-linked with formaldehyde (final concentration 1% v/v) for 10 min at room temperature, then quenched with 0.1 M glycine. Cells were pelleted at 4°C, washed three times with ice-cold PBS (pH 7.3) then frozen in liquid nitrogen and stored at –80°C. Frozen cell pellets were resuspended in 500 μl of lysis buffer (50 mM NaCl, 10 mM Tris–HCl pH 8.0, 20% w/v sucrose, 10 mM EDTA, 100 μg/ml RNase A, ¼ complete mini protease inhibitor tablet (Roche #37378900), 2000 K U/μl Ready-Lyse lysozyme (Epicentre)) and incubated at 37°C for 30 min to degrade the cell wall. 500 μl of immunoprecipitation buffer (300 mM NaCl, 100 mM Tris–HCl pH 7.0, 2% v/v Triton X-100, ¼ complete mini protease inhibitor tablet (Roche #37378900), 1 mM EDTA) was added to lyse the cells and the mixture was incubated at 37°C for a further 10 min before cooling on ice for 5 min. DNA samples were sonicated (40 A) three times for 2 min at 4°C to obtain an average fragment size of ∼500–1000 bp. Cell debris were removed by centrifugation at 4°C and the supernatant transferred to a fresh Eppendorf tube. To determine the relative amount of DNA immunoprecipitated compared to the total amount of DNA, 100 μl of supernatant was removed, treated with Pronase (0.5 mg/ml) for 60 min at 37°C then stored on ice. To immunoprecipate protein-DNA complexes, 800 μl of the remaining supernatant was incubated with rabbit polyclonal anti-DnaA, anti-DnaD and anti-DnaB antibodies (Eurogentec) for 1 h at room temperature. Protein-G Dynabeads (750 μg, Invitrogen) were equilibrated by washing with bead buffer (100 mM Na_3_PO_4_, 0.01% v/v Tween 20), resuspended in 50 μl of bead buffer, and then incubated with the sample supernatant for 1 h at room temperature. The immunoprecipated complexes were collected by applying the mixture to a magnet and washed with the following buffers for 15 min in the respective order: once in 0.5x immunoprecipitation buffer; twice in 0.5x immunoprecipitation buffer + NaCl (500 mM); once in stringent wash buffer (250 mM LiCl, 10 mM Tris–HCl pH 8.0, 0.5% v/v Tergitol-type NP-40, 0.5% w/v sodium deoxycholate 10 mM EDTA). Finally, protein-DNA complexes were washed a further three times with TET buffer (10 mM Tris–HCl pH 8.0, 1 mM EDTA, 0.01% v/v Tween 20) and resuspended in 100 μl of TE buffer (10 mM Tris–HCl pH 8.0, 1 mM EDTA). Formaldehyde crosslinks of both the immunoprecipitate and total DNA were reversed by incubation at 65°C for 16 h in the presence of 1000 U Proteinase K (excess). The reversed DNA was then removed from the magnetic beads, cleaned using QIAquick PCR Purification columns (Qiagen) and used for qPCR analysis.

### DNA scaffolds

If DNA duplexes were used, a 20 μl reaction was assembled with 10 μM of each oligonucleotide in Oligo Annealing Buffer (30 mM HEPES–KOH [pH 8], 100 mM potassium acetate and 5 mM magnesium acetate). Samples were heated in a PCR machine to 95°C for 5 min and then cooled by 1°C/min to 20°C before being held at 4°C. Double-stranded DNA complexes were purified using desthiobiotin-labelled oligonucleotides (IDT) and magnetic streptavidin beads (Dynabeads^®^ Streptavidin, Invitrogen) following annealing to a fluorescently labelled oligonucleotide (2x excess). Complexes were washed using 2x B&W buffer (10 mM Tris–HCl [pH 7.5], 1 mM EDTA and 2 M NaCl) and eluted using an equal concentration of biotin (Pierce Biotin, ThermoFisher) to that of desthiobiotin-labelled oligonucleotide prior to dilution to 1 μM in Oligo Annealing Buffer.

### Fluorescence polarisation

Fluorescein-labelled oligonucleotides were diluted on ice to 2.5 nM in DNA strand displacement buffer (10 mM HEPES–KOH [pH 7.5]; 1 mM magnesium acetate; 100 mM potassium glutamate) and 37.5 μl diluted products were dispensed in individual wells of a flat-bottom black polystyrene 96-well plate (Costar #CLS3694). DnaD proteins were diluted to 2 μM in DNA strand displacement buffer and seven to nine 2-fold dilutions were performed in the same buffer. A negative control containing no protein was used as background. Probes were allowed to equilibrate at 20°C and 12.5 μl of protein dilutions were mixed with DNA (1.875 nM final DNA concentration in 50 μl volume). Reactions were allowed to incubate for 10 min at 20°C and polarisation readings were obtained using a plate reader (BMG Clariostar) set at the same temperature. For each experiment, technical replicates were averaged and the background corresponding to each probe was subtracted from experimental values, thus reporting the specific DnaD DNA binding activity on a single substrate. Error bars indicate the standard error of the mean over two to five biological replicates.

### DNA strand separation assay

DNA scaffolds that contained one oligonucleotide labelled with BHQ2, one with Cy5 and one unlabelled (12.5 nM final concentration) were diluted in 10 mM HEPES–KOH (pH 8), 100 mM potassium glutamate, 2 mM magnesium acetate, 30% v/v glycerol, 10% v/v DMSO and 1 mM nucleotide (ADP or ATP). All the reactions were prepared on ice to ensure the stability of the DNA probe, then allowed to equilibrate at 20°C. DnaA was added to a final concentration of 650 nM to allow displacement of all the probes. DnaD and BSA were used at the same final concentration. Reactions were performed using a flat-bottom black polystyrene 96-well plate (Costar #CLS3694) in triplicate and fluorescence was detected every minute over 60 min with a plate reader (BMG Clariostar). For all reactions a negative control without protein was used as background. At each timepoint the average background value was subtracted from the experimental value, thus reporting the specific DnaA activity on a single substrate. Error bars indicate the standard error of the mean over three biological replicates.

### Phenotype analysis of origin mutants

Strains were grown for up to 72 h at 20°C or 37°C on NA plates. All experiments were independently performed at least twice and representative data are shown.

### Cryo-EM sample preparation and data collection

Four-microliter samples of purified wild-type full-length DnaD crosslinked with bis-sulfosuccinimidyl suberate (BS_3_) in the absence of DNA were applied to plasma-cleaned Quantifoil Au 2/2 200 grids, followed by plunge-freezing in liquid ethane using a Leica EM GP. Data collection was carried out at liquid nitrogen temperature on a Titan Krios microscope (Thermo Fisher Scientific) operated at an accelerating voltage of 300 kV. Micrograph movies were collected using EPU software (FEI) on a Gatan K3 detector in counting mode with a pixel size of 0.831 Å. Over 30 000 movie frames were acquired with a defocus range of approximately –0.7 to –2.7 μm. Each movie consisted of a movie stack of 50 frames with a total dose of ∼50 electrons/Å^2^ at a dose rate of 15 electrons/pixel/s.

### Cryo-EM image processing, reconstruction and model fitting

The movie stacks were aligned and summed with dose-weighting using MotionCor2 (43) via the on-the-fly processing pipeline at the Electron Bio-Imaging Centre (eBIC). Due to computational limitations only 18288 aligned micrographs (about 50% of the entire collected data) were imported into CryoSparc (v4.1.1) for further processing (44). The Contrast Transfer Function (CTF) was estimated using the patch CTF estimation function in CryoSparc, and images with poor CTF were eliminated. A small subset of 500 micrographs were used to pick the particles using the blob picker tool and extracted with a box size of 256 pixels (44). These particles were 2D classified to generate a template which was subsequently used for particle picking using the template picket tool. A total of 12 million initial particles were picked using a box size of 256 pixels and subjected to several iterative rounds of 2D classification, removing particles belonging to poor 2D template classes after each round of classification. A visual inspection of the 2D classes pointed to a clear two-fold symmetry within the particles. Several iterative rounds of 2D classification led to a final set of 1156556 particles that were used to generate three *ab-initio* models using a C2 symmetry and to classify the particles into three classes with a distribution of 31.5% (class 1), 38.4% (class 2) and 30.1% (class 3). Particles from these three *ab-initio* 3D classes were subjected to three individual homogeneous refinement processes using their respective *ab-initio* maps as starting models using C2 symmetry. Parameters with a low-resolution cut-off of 20 Å for the initial model and a 15 Å resolution starting limit to split the particles into two equal halves for convergence within the homogeneous refinement steps were used. Homogeneous refinement with particles from classes 1 and 3 did not yield a discernible model, whereas class 2 with 443 529 particles yielded a map at 6.39 Å with features corresponding to the DnaD N-terminal domain. As the DnaD^NTD^ tetramer in a dimer of dimer arrangement could be clearly observed, a subsequent homogeneous refinement step using the same set of parameters with a D2 symmetry was performed, which yielded a map at 5.47 Å with 0.143 FSC cut-off. This density map has been submitted to the Electron Microscopy Data Bank with accession code EMD-16914.

### Model building and refinement

The DnaD^NTD^ crystal structure (PDB: 2V79) was used to fit into the cryo-EM map. Two dimers could readily be docked into the map with the rigid body model fitting option in Chimera (36), which was used to generate the model of a DnaD^NTD^ tetramer. The quality of this model was check using Molprobity (45) and the coordinate file was deposited to the Protein Data Bank with entry code 8OJJ.

## RESULTS

### Residues within the DnaD^CTT^ are important for protein activity *in vivo*

It is established that DnaD has an affinity for DNA and previous studies employing protein deletions reported that this activity involves the C-terminal tail (21–23). DnaD is conserved in Firmicute pathogens and an alignment of homologous DnaD^CTT^ sequences indicated the recurrence of positively charged and aromatic residues within this region (Figure 1C). However, because alanine scanning mutagenesis did not identify single residues within the DnaD^CTT^ that were essential for DNA binding (20), we hypothesized that the DnaD^CTT^ contains a redundant set of residues capable of recognising nucleic acid substrates.

To investigate the role of charged and aromatic DnaD^CTT^ residues *in vivo*, we replaced the endogenous *dnaD* with alleles encoding for multiple alanine substitutions. Because DnaD is essential, to construct mutations at the native locus we employed an ectopic complementation system composed of an IPTG-inducible copy of *dnaD* carrying a C-terminal degradation tag (*dnaD-ssrA*) (Figure 1D). Plasmids encoding *dnaD* alanine mutants were constructed and transformed into the complementation strain of *B. subtilis*. Substitutions replacing two clusters of residues (DnaD^7A^) were required to elicit an observable growth defect as revealed by spot titre analysis (Figure 1E). While the DnaD^7A^ variant was mildly underexpressed compared to the wild-type protein (Supplementary Figure S1A), we note that a Δ*dnaD* mutant strain can sustain growth even when the level of complementing DnaD-ssrA was undetectable by immunoblot (Supplementary Figure S2). Thus, it seems unlikely that changes in expression level alone can explain the phenotype of *dnaD^7A^*.

The titration of DnaD-ssrA, showing that low protein levels could support cell growth (Supplementary Figure S2), alerted us to the possibility that leaky expression of DnaD-ssrA may contribute to the viability of *dnaD^7A^*. To examine the activity of DnaD^7A^ in the absence of DnaD-ssrA, we attempted to replace the ectopic *dnaD-ssrA* with a cassette containing *tet* (tetracycline resistance) and *bgaB* (β-galactosidase) genes, using recipient strains expressing either wild-type DnaD or DnaD^7A^ from the endogenous locus. Selection for Tet^R^ showed a ∼100-fold reduction in the number of colonies obtained in the presence of *dnaD^7A^* (Supplementary Figure S1B, C). Screening for β-galactosidase activity revealed that only a minority of the transformants observed in the presence of *dnaD^7A^* correctly integrated the *bgaB* cassette (Supplementary Figure S1B), and DNA sequencing showed that these rare blue transformants had, in fact, restored wild-type *dnaD* at the native locus. To ensure that the *dnaD^7A^* strain was genetically competent, as a control we independently performed transformations using a DNA fragment containing an antibiotic cassette that integrates at an unlinked locus. Here, a similar number of colonies were observed for each strain (Supplementary Figure S1D). Therefore, the low frequency of transformants observed when attempting to replace *dnaD-ssrA* appears specific for the *dnaD^7A^* allele, suggesting that DnaD^7A^ cannot support growth as the sole copy.

### DnaD^CTT^ mutants compromise DNA replication initiation

In *B. subtilis*, DnaD has a role in both the replication initiation and restart pathways (46–48), and was also suggested to be involved in chromosome organisation via DNA remodelling activities (26,49). To investigate potential replication defects associated with the DnaD^CTT^, we employed marker frequency analysis. Following depletion of *dnaD-ssrA* for three hours, the results showed that DnaD^7A^ cells had a significantly lower *ori:Ter* ratio, whereas DnaD variants containing fewer alanine substitutions (DnaD^4A^ and DnaD^3A^) were less affected (Figure 1F). Note that a strain harbouring *dnaD-ssrA* as the sole copy within a cell produces an *ori:Ter* ratio above 1, indicating that this residual activity may contribute to measurements in strains expressing DnaD variants (Figure 1F).

**Figure 2.**
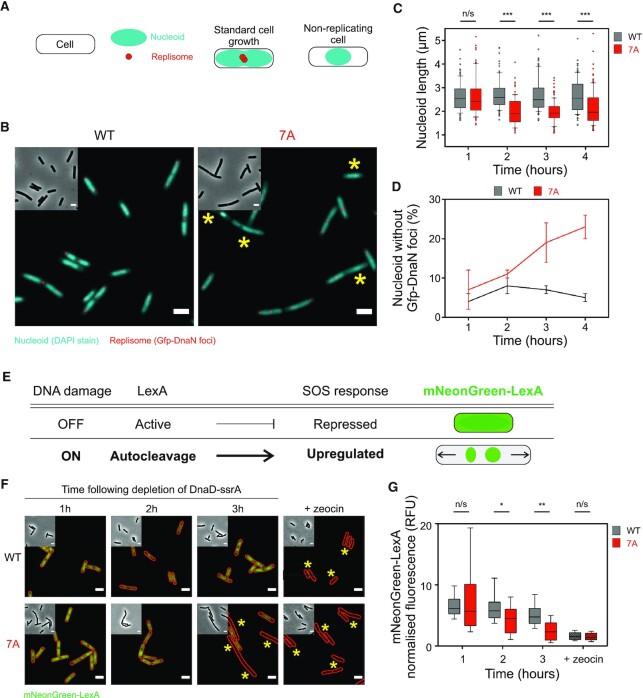
Microscopy analysis reveals that DnaD^7A^ leads to DNA replication defects. (**A**) Schematics of the active replisomes and nucleoids per cell showing the typical patterns associated with cells displaying a normal growth phenotype or non-replicating cells. (**B**) Representative images of *dnaD* strains observed by fluorescence microscopy via the system described in panel (A). Phase-contrast images are shown in the top-left corner of every image. Red dots show active replisomes (GFP-DnaN foci) and the cyan signal allows localisation of the nucleoid. Yellow stars indicate nucleoids without any GFP-DnaN foci. Wild-type corresponds to a strain encoding the ectopic *dnaD-ssrA* cassette that was depleted from IPTG and relied on the endogenous copy of wild-type *dnaD* (WT) to sustain growth (CW1144). 7A (CW1145). Scale bar indicates 2 μm. (**C**, **D**) Single-cell image analysis performed on the data obtained from experiments shown in panel (B). (**C**) shows a reduction in nucleoid size in a *dnaD^7A^* background. n/s indicates no significant difference between samples and *** significant *P*-values (*P*< 0.001) associated with a two-tailed *t*-test. (**D**) shows an increase in the number of non-replicating nucleoids in a *dnaD^7A^* background. Error bars show the standard error of the mean for two biological replicates where 100 nucleoids were analysed for each cell background. (**E**) Schematic of the DNA damage response in *B. subtilis*. The *mNeonGreen-lexA* cassette can be used as a proxy to detect induction of the SOS response following DNA damage. (**F**) Representative images of *mNeonGreen-lexA* observed by fluorescence microscopy in *dnaD* strains. Phase-contrast images are shown in the top-left corner of every image. Loss of fluorescence (mNeonGreen-LexA) indicates the presence of DNA damage and induction of the SOS response. Red outlines correspond to cell boundaries detected from phase-contrast images. Yellow stars indicate cells devoid of fluorescence. The DNA damaging reagent zeocin was used as a positive control. Wild-type corresponds to a strain encoding the ectopic *dnaD-ssrA* cassette and the endogenous copy of *dnaD* (CW1148); the 7A mutant replaces the endogenous allele (CW1149). Scale bar indicates 2 μm. (**G**) Single-cell image analysis performed on the data obtained from experiments shown in panel (**F**) showing a reduction of mNeonGreen-LexA fluorescent signal in a *dnaD^7A^* background. n/s indicates no significant difference between samples, * and ** significant *P*-values associated with a two-tailed *t*-test (*P*< 0.01 and *P*< 0.005, respectively).

To further investigate the role of DnaD^CTT^ on DNA replication, we used fluorescence microscopy to visualise chromosome content and DNA replication at the single cell level in the DnaD^7A^ strain (Figure 2A). The number of active replisomes was determined using GFP-DnaN as a reporter (50). Images were taken following depletion of the ectopically expressed DnaD-ssrA (Supplementary Figure S3A, B). Under the growth conditions used, cells containing wild-type *dnaD* at the native locus typically displayed one or more active replisomes per nucleoid (Figure 2B–D). In contrast, cells with *dnaD^7A^* at the native locus had shorter nucleoids that more frequently lacked GFP-DnaN foci (Figure 2C, D). Taken together with the marker frequency analysis, the results suggest that mutations in the DnaD^CTT^ decrease the rate of DNA replication initiation.

While analysing microscopy images, we noticed that DnaD^7A^ cells often displayed an elongated cellular morphology following DnaD-ssrA repression (Figure 2F). We speculated that because DnaD plays a role in reloading helicase following replication fork collapse and DNA repair (46,49), DnaD^7A^ might be defective in replication restart, and therefore the canonical RecA-dependent DNA damage response might be activated (51). In bacteria, RecA activation promotes auto-cleavage of the transcriptional repressor LexA (52,53), and one of the genes within the LexA regulon is the cell division inhibitor *yneA* (51). Using an endogenous *mNeonGreen-lexA* fusion as a fluorescent reporter, we observed a bright and homogeneous intracellular fluorescence signal in wild-type *dnaD* cells following DnaD-ssrA depletion (Figure 2E-G and Supplementary Figure S3D). In contrast, many DnaD^7A^ cells were observed to lack intracellular fluorescence, consistent with LexA cleavage and proteolysis (Figure 2F-G). These results suggest that DnaD^7A^ induces the RecA-dependent DNA damage response, indicating a role for DnaD after DNA replication initiation. Importantly however, we note that marker frequency analysis detected a lower *ori:Ter* ratio in the DnaD^7A^ strain, indicating that the initiation defect is the dominant phenotype.

### DnaD binds ssDNA via the DnaD^CTT^

**Figure 3.**
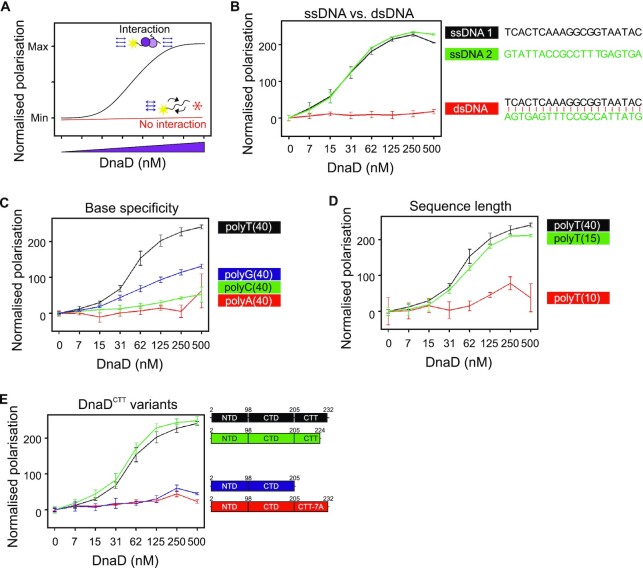
DnaD binds ssDNA through the DnaD^CTT^. (**A**) Schematics of a fluorescence polarisation assay showing interaction (black line) or no interaction (red line) between DnaD and DNA. In this assay, polarised light illuminates a protein:DNA mixture and polarisation is used as a proxy to estimate the presence of protein:DNA interactions. For a fixed concentration of fluorescein-labelled DNA, titration of DnaD produces a sigmoid curve if light is directed at samples and remains polarised at higher protein concentrations, suggesting an interaction between protein and DNA. If DNA is unbound, it tumbles rapidly upon illumination and polarised light becomes depolarised, indicating no detectable interaction between protein and DNA. A linear increase of polarisation values over titration of the protein indicates a non-specific interaction between the protein and fluorescein. (B–D) show fluorescence polarisation analyses of wild-type DnaD incubated with a range of DNA substrates. (**B**) shows that DnaD preferentially binds ssDNA over dsDNA. Sequences were randomly generated and the DNA duplex (dsDNA) corresponds to the annealing of individual ssDNA sequences. ssDNA 1 (oCW1036), ssDNA 2 (oCW1124) and dsDNA (oCW1034:oCW1124). (**C**) shows that DnaD preferentially binds thymidine bases. The protein was incubated with homopolymer sequences of a size of 40 nucleotides. polyT(40) (oCW837), polyG(40) (oCW854), polyC(40) (oCW852), polyA(40) (oCW853). (**D**) shows that DnaD preferentially binds substrates of a size equal to or over 15 nucleotides. Substrates were chosen based on the binding preference observed in panel (C). polyT(40) (oCW837), polyT(15) (oCW1029), polyT(10) (oCW834). (**E**) Fluorescence polarisation analysis of DnaD protein variants indicates that DnaD residues 205–224 are important of DNA binding activity and that the DnaD^CTT^ variant DnaD^7A^ is defective in ssDNA binding. All proteins were incubated with a homopolymer of 40 thymidine bases (oCW837). The black line corresponds to wild-type DnaD, the green line to a variant lacking the last eight amino acid residues of the DnaD^CTT^, the blue line to a variant lacking the entire DnaD^CTT^ and the red line to the DnaD^7A^ mutant where multiple alanine substitutions were targeted at positively charged and aromatic residues of the DnaD^CTT^. Error bars in panels (B-E) indicate the standard error of the mean for 2–4 biological replicates.

To directly investigate the DNA binding activity of DnaD *in vitro*, we established a fluorescence polarization assay to detect the interaction of DnaD with fluorescein labelled DNA substrates (Figure 3A) (54). It was found that wild-type DnaD (i) binds ssDNA with a higher affinity than dsDNA (Figure 3B), (ii) displays a preference for polythymidine (Figure 3C), and (iii) requires a ssDNA substrate of more than 10 nucleotides in size (Figure 3D).

Next, DnaD variants with alterations to the C-terminal tail were purified: DnaD^7A^ and two truncations, which removed either the DnaB interaction region (DnaD^1-224^ (20)) or the entire C-terminal tail containing the putative ssDNA binding residues (DnaD^1-205^). Both DnaD^7A^ and DnaD^1-205^ were unable to interact with a fluorescently labelled dT_40_ substrate, whereas DnaD^1-224^ retained activity similar to wild-type (Figure 3E). Size exclusion chromatography (SEC) followed by multiple-angle light scattering (MALS) showed that DnaD^1-205^, the largest truncation, remained a tetramer (Supplementary Figure S4A–C). We also verified using SEC that DnaD^7A^ and DnaD^1-224^ assembled into tetramers (Supplementary Figure S4D). These results suggest that DnaD binds ssDNA via its DnaD^CTT^.

### DnaD interacts with a specific single-strand DNA binding element within the unwinding region of *oriC*

**Figure 4.**
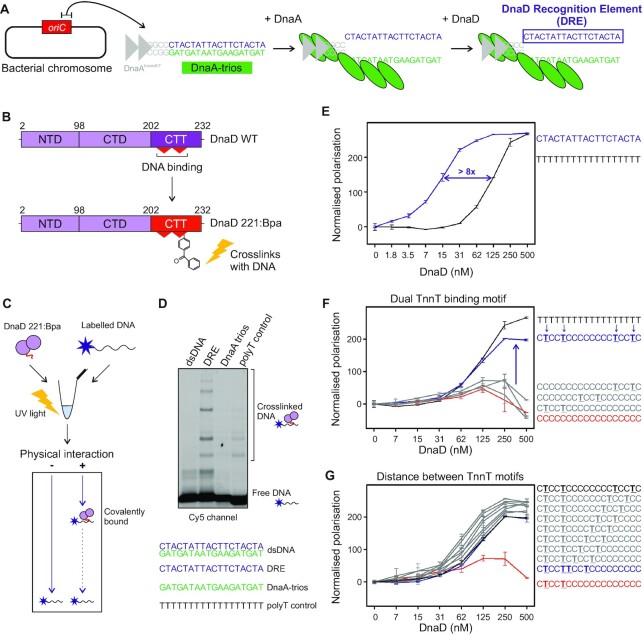
DnaD binds to the sequence facing the DnaA trios. (**A**) Illustration of the proposed basal origin unwinding mechanism in *B. subtilis*. The closed origin region *incC* is bound by DnaA on DnaA boxes, which leads to partial melting of the origin and strand separation via DnaA oligomer formation on the DnaA-trios. The complementary sequence to the DnaA-trios is proposed to be a specific binding substrate for DnaD. (**B**) DnaD primary structure schematics showing the incorporation of a non-natural amino acid residue within DnaD^CTT^ to probe physical interactions between DnaD and DNA. As opposed to the wild-type protein (WT), DnaD^K221Bpa^ can be crosslinked to DNA if the residue DnaD^221^ comes in contact with nucleic acids. NTD denote the N-Terminal Domain, CTD the C-Terminal Domain and CTT the C-Terminal Tail of DnaD. (**C**) Schematics of the Bpa crosslinking assay used to probe direct interactions between DnaD and DNA. DnaD^K221Bpa^ is incubated with a labelled oligonucleotide and reactions are crosslinked using UV exposure before running products on a denaturing gel. Following electrophoresis, free DNA runs to the bottom of the gel and the migration of covalently bound DNA:protein complexes is delayed. (**D**) Bpa crosslinking assay showing that DnaD specifically interacts with the DRE via the amino acid residue 221 in the DnaD^CTT^. Incubation with Cy-5 labelled oligonucleotides shows that DnaD interacts with ssDNA and has greater binding affinity to the DRE compared to the DnaD trios. Oligonucleotide sequences are indicated below the gel: dsDNA (oSP1132:oCW1040), DRE (oSP1132), DnaA trios (oSP1133), polyT control (oSP1135). (E–G) show fluorescence polarisation analyses of wild-type DnaD incubated with a range of DNA substrates. (**E**) shows that DnaD has significantly higher affinity for the DRE sequence than a polyT substrate of the same size. The purple line shows binding to the DRE (oCW1039) and black line binding to a polyT substrate (oCW1088). (**F**) shows that DnaD requires at least two TnnT elements to bind ssDNA. The black line shows binding to a polyT18 substrate (oCW1088), the red line shows incubation with a polyC18 substrate (oCW1089), the blue line indicates binding to a polyC substrate with two TnnT elements (oCW1128), and grey lines (from top to bottom) show incubation profiles with three oligonucleotides containing a single TnnT element (oCW1165, oCW1140 and oCW1164, respectively). (**G**) shows that the distance separating two TnnT elements does not affect the ability of DnaD to bind ssDNA. The black line shows binding to a polyC substrate with two TnnT elements separated by a distance of eight nucleotides (oCW1128). Grey lines show binding of DnaD to various ssDNA sequences where the distance separating TnnT repeats in the context of a polyC substrate is gradually reduced (from top to bottom oCW1491, oCW1492, oCW1493, oCW1494, oCW1495, oCW1496 and oCW1497) down to juxtaposition of the two TnnT repeats (blue line, oCW1498). The red line shows the incubation profile of DnaD with a substrate containing only one TnnT element (oCW1164). Error bars in panels (E–G) indicate the standard error of the mean for 2–6 biological replicates.

It has been found that DnaD requires a direct interaction with DnaA to be recruited to *oriC* (20). We speculated that this protein:protein interaction could guide DnaD to the *B. subtilis* chromosome origin for it to bind DNA (Figure 4A) (55). To test for a direct physical interaction between the DnaD^CTT^ and *oriC*, we used protein:DNA photo-crosslinking. Here the non-natural photoactivatable amino acid p-benzoyl-L-phenylalanine (Bpa) was incorporated at the 221^st^ amino acid (DnaD^K221Bpa^, Figures 1C, 4B-C) using an engineered amber suppressor tRNA with its cognate aminoacyl tRNA synthase (39). To detect DnaD:DNA crosslinking following UV irradiation, oligonucleotide substrates were fluorescently labelled and nucleoprotein complexes were separated by size using SDS-PAGE.

**Figure 5.**
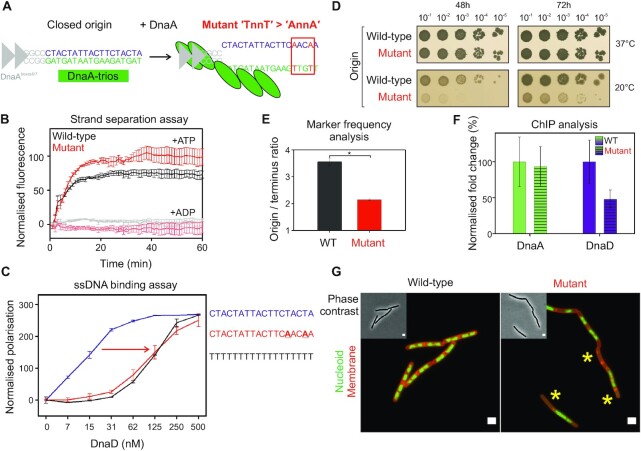
The DRE promotes specific ssDNA binding activity of DnaD *in vitro* and is required for efficient DNA replication initiation *in vivo*. (**A**) Schematics of *B. subtilis* origin unwinding region showing the potential impact of a mutation disrupting the distal 5′-TnnT-3′ element of the DRE with respect to DnaA boxes. (**B**) Strand separation assay showing that DnaA is comparably able to unwind substrates mimicking the wild-type origin or an origin where the distal DnaA trios have been disrupted as shown in panel (A). Background corresponding to the basal fluorescence of DNA complexes was subtracted from the curves. Grey and pink lines respectively show that DnaA does not unwind wild-type or mutant sequences in the presence of ADP. Black and red lines show that DnaA can unwind both wild-type and mutant origin complexes in the presence of ATP. Error bars indicate the standard error of the mean for 3–4 biological replicates. DNA substrate: oHM558/oHM778/oHM590. (**C**) Fluorescence polarisation analysis showing that a decrease in DnaD binding affinity to a DRE mutant where a 5′-TnnT-3′ motif has been mutated. The purple line (oCW1039) shows binding of DnaD to a sequence corresponding to the DRE. The red line (oCW1247) indicates that DnaD binding to a substrate where a 5′-TnnT-3′ element has been mutated is comparable to the profile of a same size polyT sequence (black line, oCW1088). (**D**) Spot titre analysis of the DRE mutant from panel (A) shows that introducing this mutation *in vivo* confers a cold-sensitive phenotype. Wild-type (168CA), Mutant (CW691). (**E**) Marker frequency analysis of the DRE mutant shown in panel (D) indicates that cells harbouring this mutation are impaired in DNA replication initiation at 20°C as measured by quantitative PCR. * shows a *P*-value of 0.018. Error bars show the standard error of the mean for three biological replicates. WT (168CA), Mutant (CW691). (**F**) ChIP analysis showing that the DRE mutant impairs the recruitment of DnaD to the origin. While DnaA is similarly recruited to the wild-type or mutant origins, DnaD was significantly less enriched in a background where a 5′-TnnT-3′ element of the DRE was mutated. The normalised fold change corresponds to the fold change in %IP observed for recruitment of DnaA and DnaD at the origin compared to background. Experiments were performed at 37°C and 100% indicates the reference fold change observed in wild-type cells. * shows a *P*-value of 0.0399 and n/s a non-significant difference. WT (168CA), Mutant (CW691). Primers used for the origin in panels (F-G) annealed within the *incC* region. Error bars show the propagated standard error of the mean for 3 biological replicates. (**G**) Representative microscopy images show that the mutation impairing the DRE leads to the appearance of anucleated cells at 20°C. Green fluorescence corresponds to imaging of the nucleoid by DAPI staining and red fluorescence depicts cellular membranes (Nile red). Wild-type (168CA), Mutant (CW691). Scale bar indicates 2 μm.

A clear set of higher molecular weight species was detected when the strand complementing the DnaA-trios was incubated with DnaD^K221Bpa^ (Figure 5D). Annealing this strand with the DnaA-trios to form a dsDNA substrate abolished UV crosslinking (Figure 4D), consistent with the model that DnaD binds ssDNA (Figure 3B). Moreover, DnaD^K221Bpa^ crosslinking to this strand appeared most efficient, compared to ssDNA substrates with either DnaA-trios or a homopolymeric sequence (Figure 5D, Supplementary Figure S5). Treatment with proteinase K degraded the higher molecular weight species, indicating that these are bonafide nucleoprotein complexes (Supplementary Figure S5B). Finally, fluorescence polarization confirmed that DnaD binds with highest affinity to the strand complementing the DnaA-trios (Figure 4E, Supplementary Figure S6A). Based on these results and combined with the observation that DnaD binds the dT_18_ substrate better than other homopolymeric ssDNA (Supplementary Figures S5B and S6A), we hypothesized that thymidine might be the major specificity determinant within the preferentially bound strand.

To identify putative DnaD binding motifs within ssDNA, thymidine bases were systematically introduced within dC_18_ or dA_18_ substrates and DnaD binding was assessed using fluorescence polarization. The results indicate that two motifs of 5′-TnnT-3′ are necessary and sufficient for DnaD to associate specifically with ssDNA (Figure 4F and Supplementary Figure S6B–D). Intriguingly, the distance between the two 5′-TnnT-3′ motifs can be varied widely (Figure 4G). This is consistent with the multiple higher molecular weight species detected by UV crosslinking (Figure 4D), as this substrate contains five potential 5′-TnnT-3′ motifs. To further understand the specificity of DnaD binding ssDNA, either cytosines or adenines were introduced into a dT_18_ substrate at positions corresponding to the native sequence complementing the DnaA-trios. Using these fluorescently labelled substrates in polarization assays, an increase in DnaD binding affinity was observed when cytosines were present, but not with adenines (Supplementary Figure S6E). Even so, because none of these substrates are recognised by DnaD as well as the wild-type sequence (Supplementary Figure S6E), it appears that all three nucleobases are critical. Based on these properties, we have termed the ssDNA sequence complementary to the DnaA-trios the *D*naD *R*ecognition *E*lement (DRE), and we propose that pairs of 5′-TnnT-3′ motifs form the core of the DnaD binding site.

It has been observed that tetramerisation is crucial for DnaD function *in vivo*, and amino acid substitutions have been identified that specifically disrupt either dimerization (DnaD^L22A^) or tetramerization (DnaD^F6A^) (20). Using a minimal substrate harbouring two 5′-TnnT-3′ motifs, neither the monomeric DnaD^L22A^ nor the dimeric DnaD^F6A^ variant were able to interact with this substrate as well as the wild-type DnaD protein (Supplementary Figure S6F). These results suggest that DnaD requires tetramerisation to bind specific elements of the origin, which supports previous results showing that a DnaD tetramer is essential *in vivo* (20).

### The DRE is required for recruiting DnaD to the chromosome origin

We next wanted to determine the role of the DRE *in vivo*. However, the DRE and the DnaA-trios are inherently linked ssDNA binding motifs that we propose are bound by DnaD and DnaA, respectively. Therefore, we set out to identify separation of function mutations within this region that support DnaA activities while disrupting DnaD binding.

Previous studies have suggested that the DnaA-trios proximal to the DnaA-boxes are the most critical for DnaA unwinding activity (16–18,56). Therefore, we hypothesized that mutating the 5′-TnnT-3′ motif distal to the DnaA-boxes might preferentially inhibit DnaD binding while leaving DnaA activity relatively unperturbed. Consistent with this notion, it was found that DnaA DNA strand separation activity *in vitro* was similar to wild-type when the distal 5′-TnnT-3′ motif was changed to 5′-AnnA-3′ (Figure 5A, B). Relatedly, we found that DnaD alone does not promote DNA strand separation (Supplementary Figure S7A–C), nor does it specifically stimulate DnaA DNA strand separation activity (Supplementary Figure S7D, E). In contrast to the unperturbed DnaA strand separation activity on *oriC* substrates where the distal 5′-TnnT-3′ motif was changed to 5′-AnnA-3′, fluorescence polarisation *in vitro* revealed that these mutations significantly decreased DnaD ssDNA binding (Figure 5C). Together, the data suggests that disrupting the 5′-TnnT-3′ motif distal to the DnaA-boxes specifically impairs DnaD.

Next, we engineered a strain with the distal 5′-TnnT-3′ motif mutated to 5′-AnnA-3′ (5′-CTACTATTACTTCTACTA-3′ → 5′-CTACTATTACTTCAACAA-3′). It was observed that this mutant displays a growth defect at 20**°**C (Figure 5D) and marker frequency analysis showed that the strain has a significantly lower DNA replication initiation frequency compared to wild-type cells at the lower temperature (Figure 5E). Importantly, ChIP showed that DnaD recruitment to *oriC* was specifically reduced in the origin mutant, whereas the recruitment of DnaA was relatively unaffected (Figure 5F and Supplementary Figure S8A–C). Consistent with a replication initiation defect (57–59), fluorescence microscopy revealed that mutant cells had abnormal chromosome content, including an increase in cells lacking DNA at the restrictive temperature (Figure 5G and Supplementary Figure S8D). In contrast to DnaD^7A^, cell division was not blocked in the *oriC* mutant and there was no degradation of the mNeonGreen-LexA DNA damage reporter (Supplementary Figure S8E, F). Taken together, the results are consistent with the DRE functioning as a specific ssDNA binding site for DnaD within the *B. subtilis* chromosome origin unwinding region.

### Architecture of a DnaD tetramer determined by cryo-electron microscopy

**Figure 6.**
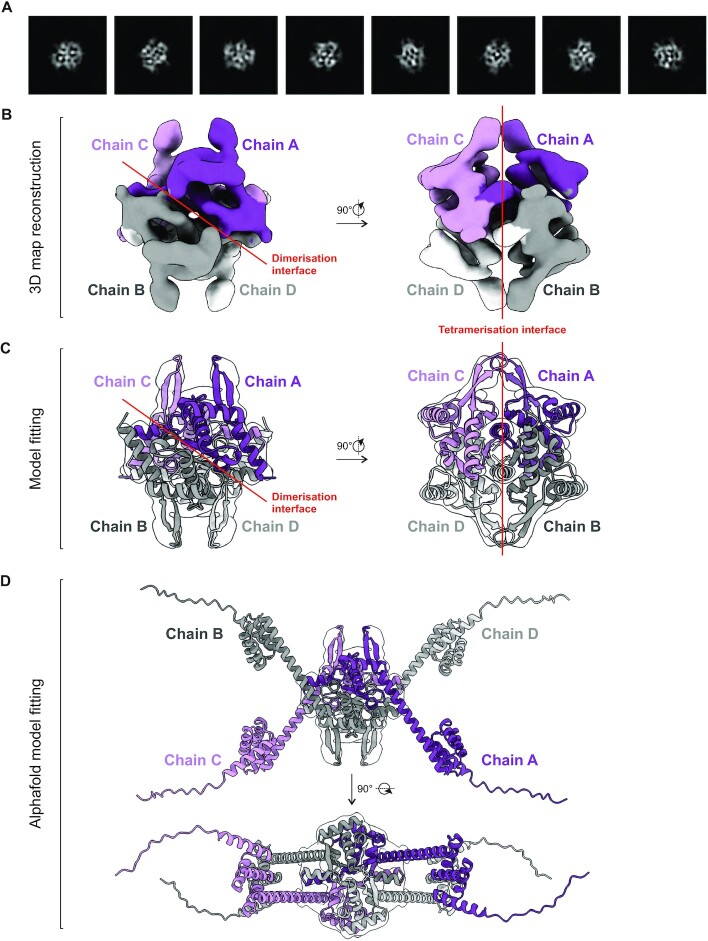
Cryo-EM structure of a DnaD tetramer. (**A**) Representative 2D classes of DnaD^NTD^ observed by cryo-EM. (**B**) Cryo-EM map of the DnaD^NTD^ tetramer shown 90° apart. (**C**) Cryo-EM density map shown in panel (B) fitted with the available DnaD^NTD^ substructure (PDB: 2V79). The maps in (B, C) are coloured based on the individual chains and depict dimerisation/tetramerisation interfaces. (**D**) The full-length DnaD model produced with Alphafold docked into the cryo-EM map of the DnaD^NTD^ tetramer. The four individual chains A, B, C and D are coloured in purple, grey, light grey and pink, respectively.

The structure of the DnaD^CTT^ has not been observed experimentally. In an attempt to place the DnaD^CTT^ within the DnaD tetramer, we characterized the full-length protein using single particle cryo-electron microscopy (cryo-EM, Supplementary Table S4). Complexes were prepared in the absence or presence of crosslinker (BS_3_ or formaldehyde), either with or without ssDNA (DRE or polyT substrates). Reactions derived from BS_3_-crosslinked DnaD in the absence of DNA produced the best samples for imaging. Data analysis from 2D classes (Figure 6A) and image processing yielded a 5.5 Å map fitting the DnaD^NTD^ assembled into a homotetramer (Figure 6B, C and Supplementary Figure S9). Alphafold (60) was used to generate a full-length model of a DnaD tetramer from the cryo-EM structure (Figure 6D).

Previously, an alanine scan of DnaD was performed to identify residues important for protein function (20). Mapping positions of alanine substitutions that conferred a growth defect onto the model revealed that the majority clustered together at either the dimerisation or tetramerisation interfaces (Supplementary Figures S10 and S11, respectively). In particular, a surface of aromatic residues assembled by a DnaD dimer (F6 and W103/Y107 from each monomer) is observed to create a tetramerisation interface. It was also noted that essential residues required for interacting with DnaA were found to run along one extended surface formed by the DnaD tetramer (Supplementary Figure S12). Together, these observations support the physiological relevance of the DnaD^NTD^ structure.

The Alphafold model suggests that the DnaD^CTD^ and DnaD^CTT^ extend away from the DnaD^NTD^ tetramer core. Interestingly, in this arrangement the two DnaD^CTT^ of a dimer point in opposite directions (Figure 6C, D), while formation of a DnaD tetramer places two DnaD^CTT^ (i.e. one from each dimer) into close proximity (Figure 6C). This model is compatible with several observations regarding DnaD binding ssDNA: (i) two TnnT motifs are required (Figure 4F); (ii) the position of two TnnT motifs is not fixed (Figure 4G); (iii) the DnaD dimer is defective for specific ssDNA binding (Supplementary Figure S6F).

## DISCUSSION

**Figure 7.**
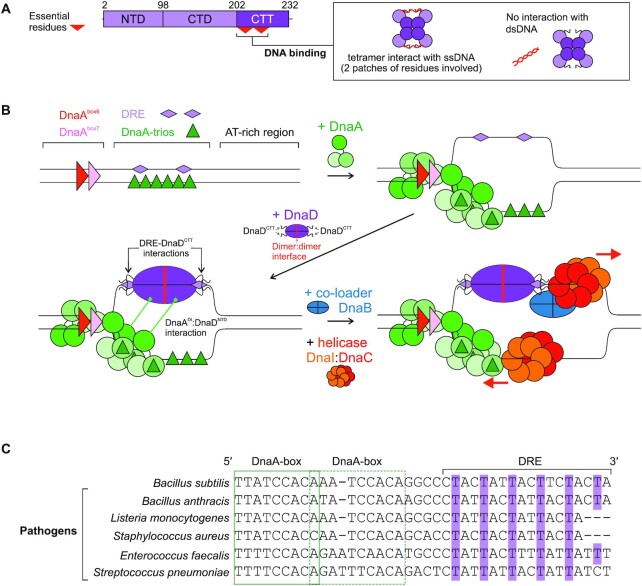
The complex of DnaD with the DRE provides a potential mechanism for strand specific helicase loading. (**A**) DnaD mutations identified two patches of residues in the DnaD^CTT^ that are critical for ssDNA binding. (**B**) Model of the chromosomal replication initiation pathway in *B. subtilis*. DnaA binds DnaA-boxes in the *incC* region, leading to oligomer formation on DnaA-trios and initial opening of the origin. DnaD is then recruited and loaded on the exposed top strand (DRE) through both DRE:DnaD^CTT^, as well as interactions between DnaA domain I and the DnaD N-terminal domain, which acts as a scaffold for further recruitment of the co-loader protein DnaB and bidirectional loading of the helicase complex DnaI:DnaC. DnaD C-terminal tail (DnaD^CTT^), DnaA domain I (DnaA^DI^), DnaD N-terminal domain (DnaD^NTD^). (**C**) Nucleotide alignment of DRE sequences showing the regular spacing and preservation of 5′-TnnT-3′ repeats (thymidines highlighted in purple) within the origins of pathogens encoding *dnaD* in their genomes. The relative position of proximal DnaA-boxes is indicated by green outlines.

Here we characterised the DNA binding activities of the essential *B. subtilis* DNA replication initiation protein DnaD. We found that DnaD binds a new ssDNA motif (DRE) within the *B. subtilis* chromosome origin via the DnaD^CTT^ (summarized in Figure 7A-B). Based on the findings that DnaD tetramerization is required for maximal ssDNA binding (Figure 3E) and on the structural model for DnaD^CTT^ localisation (Figure 6D), we speculate that at *oriC* each DnaD dimer (within a tetramer) donates one DnaD^CTT^ to engage the DRE (Figure 7B).

While this work has focused on the ssDNA binding activity of DnaD at *oriC*, DnaD also has an established role during helicase loading at a collapsed replication fork (46,47). We note that strains expressing DnaD^7A^ displayed a cell elongation phenotype consistent with arrest of cytokinesis (Figure 2F), in contrast with mutations in the DRE (Supplementary Figure S8E). We speculate that DnaD needs to engage ssDNA during replication restart and that failure to bind stably delays helicase loading, potentially exposing ssDNA regions that can be bound by RecA and stimulating the SOS response (even more so if chromosome breaks are the cause of replisome collapse). An alternative explanation is that the DnaD^CTT^ is required to interact with PriA, the initiator protein for the replication restart pathway. However, because direct interactions between PriA and DnaD have not been detected (61), the available data currently favours the former hypothesis.

### A mechanism for bidirectional DNA replication at a bacterial chromosome origin

A model was proposed for helicase loading at one end of a DnaA oligomer, where the AAA+ class of helicase loader engages the AAA+ motif of DnaA and guides deposition of helicase onto ssDNA (Figure 7B) (19). This mechanism results in helicase loading around ssDNA in the correct orientation for 5′→3′ translocation through *oriC*. In *B. subtilis*, this would correspond to helicase encircling the strand encoding DnaA-trios (Figure 7B).

In contrast, the mechanism for loading a helicase onto the complementary strand was unclear. In *E. coli*, biochemical and genetic assays have suggested that DnaA domain I interacts directly with the helicase (62,63), and it has been shown that DnaA^E21^ is essential for viability and required for helicase loading *in vitro* (64). In a recent study in *B. subtilis*, we identified residues in DnaA^DI^ that are essential for cell viability and required for the direct recruitment of DnaD to *oriC* (20). Generalizing, it appears that DnaA^DI^ acts as a critical protein interaction hub involved in helicase recruitment in diverse bacterial species (20,65), albeit through mechanisms involving distinct partners. Importantly however, these protein:protein interactions alone do not resolve how a second helicase is recruited to a specific DNA strand with the correct geometry to support bidirectional replication.

Many bacterial chromosome origins encode a core set of sequence elements that direct DnaA oligomerization onto ssDNA, thus dictating the strand onto which the AAA+ helicase chaperone would dock (18). Here we report that the DRE, located opposite to where the DnaA oligomer binds, could provide a mechanism for orchestrating strand-specific DnaD recruitment in *B. subtilis*; this mode of action is potentially conserved in many Firmicutes including the pathogens *Staphylococcus, Streptococcus, Enterococcus* and *Listeria* (Figure 7C) (66). Considered together with studies of primosome assembly at a single-strand origin, where binding of DnaD to ssDNA promotes subsequent helicase loading (Supplementary Figure S13) (67), we propose that the specific interaction of DnaD with the DRE provides a pathway for loading a second helicase to support bidirectional DNA replication (Figure 7B). Based on the relatively poor interaction of DnaD with dsDNA (Figures 3B, 4D), we propose that DnaD engages the DRE following DNA strand separation by DnaA. These results further delineate a step-wise assembly pathway of DnaA and DnaD at the *B. subtilis* chromosome origin.

### Outstanding questions for helicase loading mechanisms

Here we propose a mechanism to orchestrate bidirectional helicase recruitment at the *B. subtilis* chromosome origin. This raises many new questions for helicase loading in *B. subtilis*, such as how DnaD (along with DnaA and DnaB) orientates the DnaI:helicase complex onto the strand encoding the DRE. We speculate that within the open complex formed by initiation proteins and dsDNA/ssDNA at *oriC*, the distal junction between dsDNA and the unwound ssDNA could physically influence the subsequent events. Additionally, protein:protein interactions at *oriC* nucleoprotein complexes involving DnaA, DnaD and DnaB could play roles.

How is the temporal loading of two helicases orchestrated at *oriC*? Studies of *E. coli* helicase loading onto artificial DNA scaffolds that mimic an open chromosome origin indicated that DnaA preferentially recruits helicase onto the strand corresponding to where the DRE is located (68). Whether this order of recruitment holds during the physiological helicase loading reaction is unclear. While we favour a model where loading of the two helicases at *oriC* is reproducibly sequential, an alternative hypothesis is that loading of the two helicases is stochastic.

Are there other sequence elements within *oriC* that direct helicase loading? The discovery of the DnaA-trios and the DRE indicate that bacterial chromosome origins encode more information than previously appreciated. We note that many chromosome origins contain an intrinsically unstable AT-rich region (55,69) where one of the helicases is loaded *in vitro* (70). The relative redundancy of dual 5′-TnnT-3′ repeats and the flexible distance separating these elements (Figure 4G) supports the notion that additional sequence dependent information may be located within these AT-rich sites, or elsewhere. Further characterization of the nucleoprotein complexes formed at *oriC*, as well as dissection of downstream helicase loader proteins, will be needed to provide answers.

What about bacteria lacking a *dnaD* homolog (71,72)? One hypothesis is that DnaA domain I itself could bind ssDNA and promote helicase loading. The structure of DnaA domain I is similar to that of the K homology (KH) domain (64), which is often observed as a module for binding single-stranded nucleic acids. In *E. coli* it has been reported DnaA domain I alone both specifically interacts with ssDNA from *oriC* and directly contacts the replicative helicase (62–64). We also speculate that there may be species specific ssDNA binding proteins that could act analogously to DnaD. Supporting this notion, the surface of DnaA domain I that interacts with DnaD appears to be the same surface used for unrelated proteins to bind DnaA in other organisms (73–76).

## DATA AVAILABILITY

All plasmids and strains are available upon request. Microscopy data reported in this paper will be shared upon request. The atomic coordinate file has been deposited at the Protein Data Bank with accession code 8OJJ. The cryo-EM density map has been deposited at the Electron Microscopy Data Bank with accession code EMD-16914.

## Supplementary Material

gkad277_Supplemental_Files

## Notes

Present address: Simone Pelliciari, MRC Human Genetics Unit, Institute of Genetics and Cancer, University of Edinburgh, Western General Hospital, Edinburgh, UK.

Present address: Elie Marchand, Research Unit in Biology of Microorganisms, Department of Biology, Université de Namur, Namur, Belgium.
