# Supplementary material for: The DNA replication initiation protein DnaD recognises a specific strand of the Bacillus subtilis chromosome origin
Source: Nucleic Acids Res. 2023 Apr 24;51(9):4322–40. doi: 10.1093/nar/gkad277 (PMC10201434; doi:10.1093/nar/gkad277)
Supplement: gkad277_Supplemental_Files [file gkad277_supplemental_files.zip › DnaD-DRE_supplementary-figures_proof.pdf]

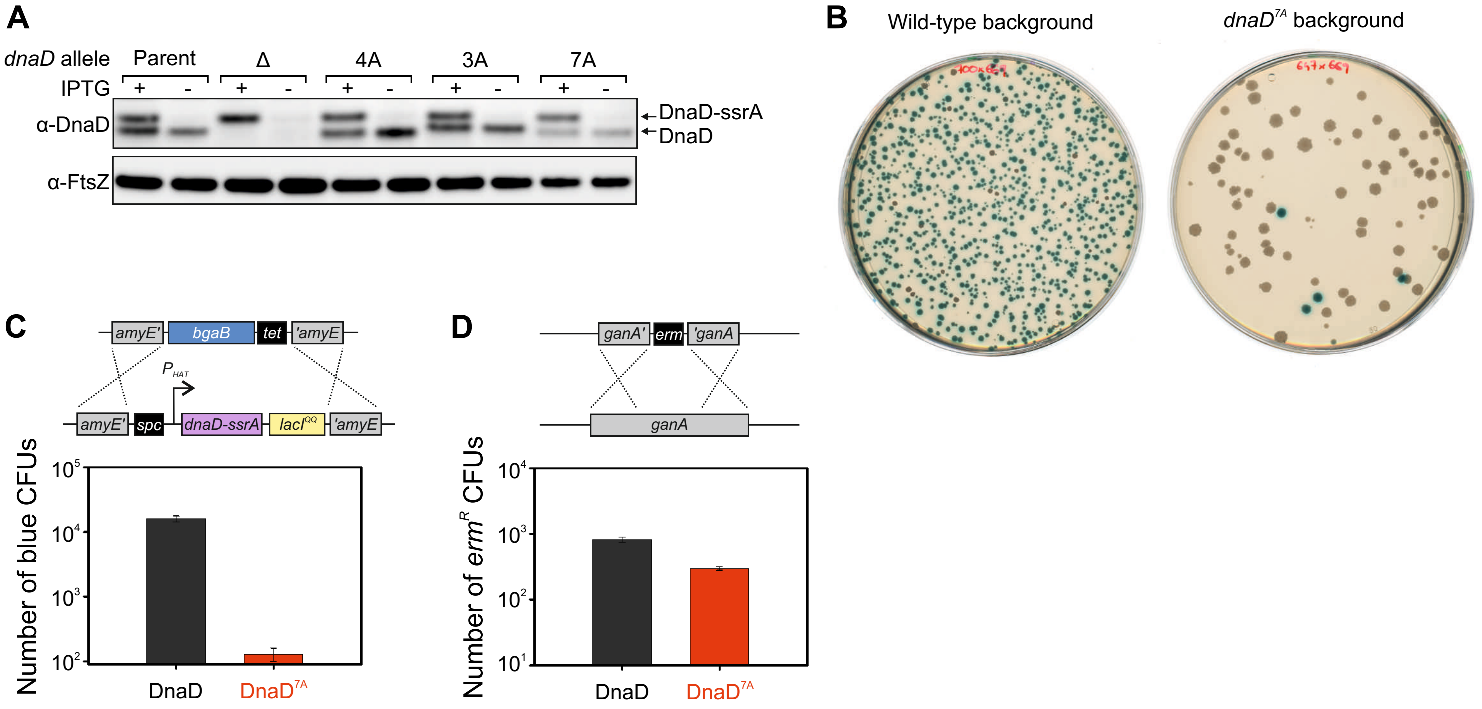

Figure S1. DnaD^{7A} does not sustain growth. (A) Immunobloting of multiple alanine substitution DnaD variants targeting positively charged and aromatic residues within the DnaD^{CTT}. The presence of IPTG shows expression of the ectopic DnaD-SsrA used for complementation. The tubulin homolog FtsZ was used as a loading control. Parent (Cw162), Δ (CW197), 4A (CW412), 3A (CW415), 7A (CW647). **(B)** Transformation plate showing a decrease in the number of blue colonies obtained in the *dnaD^{7A}* background in an attempt to knock-out *dnaD-ssrA*. **(C)** Quantification of the number of blue colony forming units (CFUs) obtained after attempting to remove the *dnaD-ssrA* cassette from a strain carrying wild-type DnaD or the 7A allele at the endogenous locus. Sequencing of eventual blue transformants in the *dnaD^{7A}* background revealed that wild-type *dnaD* was restored. Recipient strains: DnaD (CW162), DnaD^{7A} (CW647). **(D)** Control DNA showing that strains transformed in panel (B) are both competent and integrated an *erm* cassette (*erm^R* CFUs) at the ectopic locus *ganA* at a similar rate.

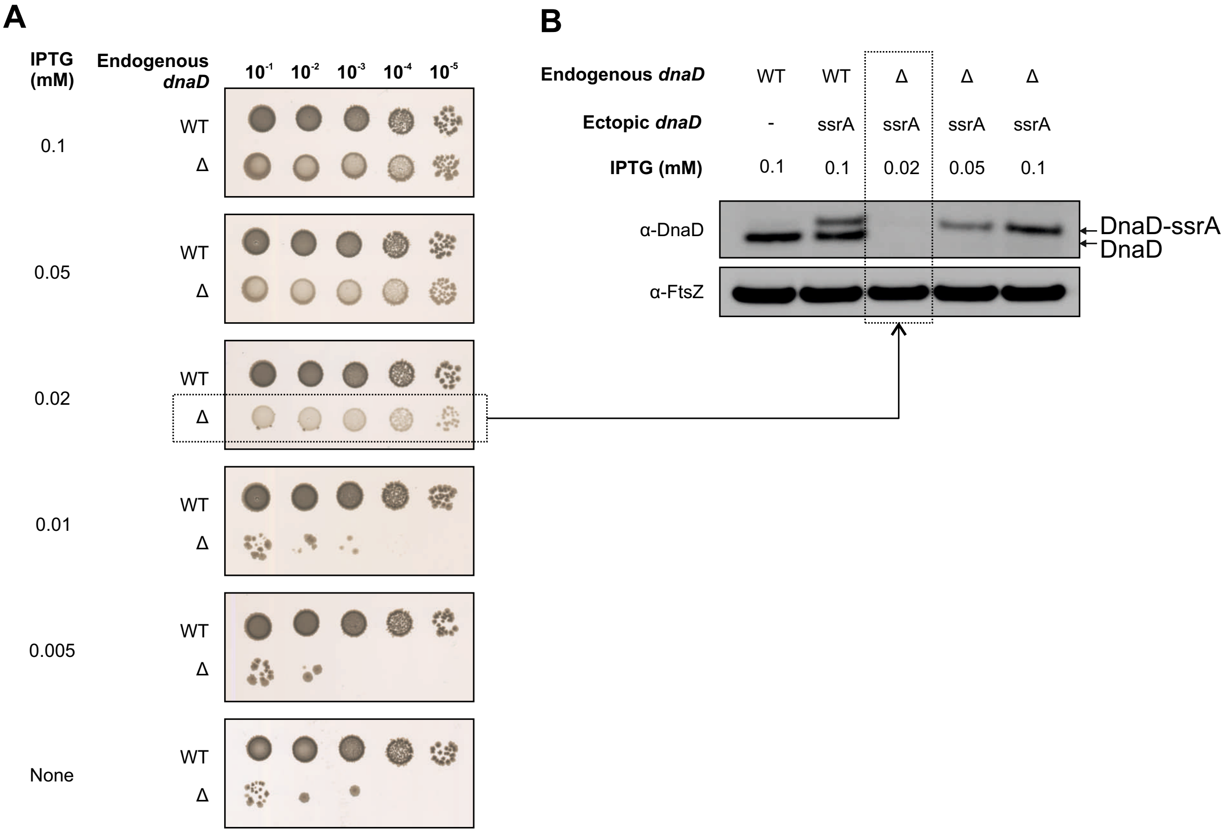

Figure S2. Low levels of DnaD expression sustain cell growth. (A) DnaD-SsrA was titrated via IPTG induction. The *dnaD-ssrA* cassette was able to sustain growth at IPTG concentration of 0.02 mM and above. WT (CW162), Δ (CW197). **(B)** Immunobloting shows that expression of DnaD was undetectable in viable colonies grown with 0.02 mM IPTG. The tubulin homolog FtsZ was used as a loading control.

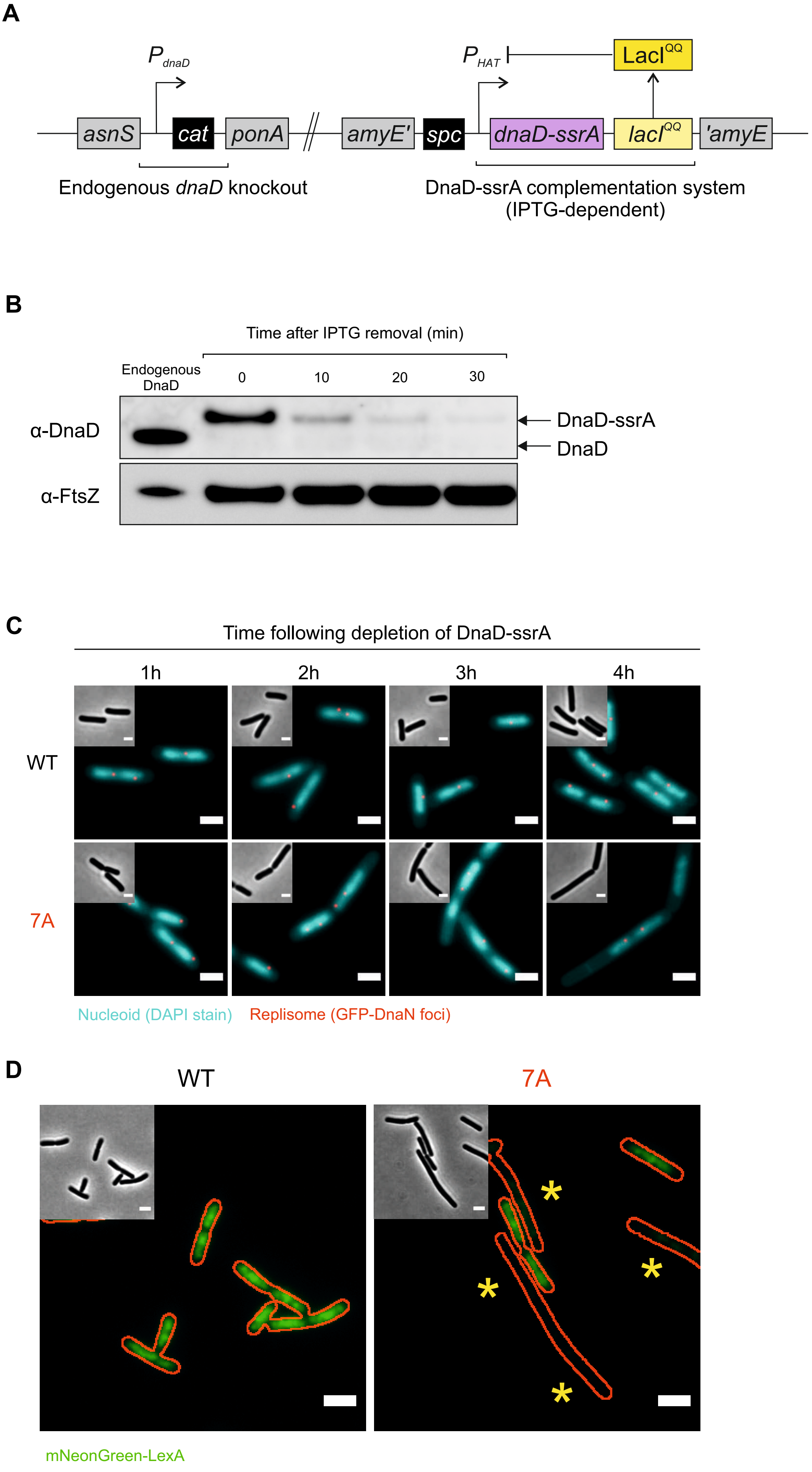
A
P_{dnaD}
P_{HAT}
LacI^{QQ}
asnS
cat
ponA
amyE'
spc
dnaD-ssrA
lacI^{QQ}
'amyE
Endogenous dnaD knockout
DnaD-ssrA complementation system
(IPTG-dependent)
B
Time after IPTG removal (min)
Endogenous DnaD
0
10
20
30
α-DnaD
α-FtsZ
DnaD-ssrA
DnaD
C
Time following depletion of DnaD-ssrA
1h
2h
3h
4h
WT
7A
Nucleoid (DAPI stain)
Replisome (GFP-DnaN foci)
D
WT
7A
*
mNeonGreen-LexA

Figure S3. Depletion of DnaD-ssrA *in vivo* reveals aberrant chromosome replication in a DnaD^{7A} background. (A) Schematics of the inducible system used to investigate the expression of the *dnaD-ssrA* fusion and degradation of DnaD-ssrA upon depletion of IPTG. In these cells (Cw164), the ectopic *dnaD-ssrA* fusion is the only copy of *dnaD* available to sustain growth in the presence of inducer (IPTG) because the endogenous *dnaD* operon was replaced by a chloramphenicol antibiotic resistance cassette (*cat* gene). **(B)** Immunoblot analysis of the inducible *dnaD-ssrA* cassette showing that degradation of DnaD-SsrA is achieved in about 30 minutes post-depletion of IPTG (CW164); endogenous *dnaD* indicates expression of wild-type DnaD in *B. subtilis* 168CA. Detection of the tubulin homolog FtsZ was used as a loading control. **(C)** Representative images of *dnaD* strains observed by fluorescence microscopy via the system described in Figure 2A. Phase-contrast images are shown in the top-left corner of every image. Red dots show active replisomes (GFP-DnaN foci) and the cyan signal allows localisation of the nucleoid. Wild-type corresponds to a strain encoding the ectopic *dnaD-ssrA* cassette that was depleted from IPTG and relied on the endogenous copy of wild-type *dnaD* (WT) to sustain growth (CW1144). 7A (CW1145). **(D)** Representative fluorescence microscopy images of *dnaD* strains showing activation of the SOS response in a DnaD 7A background. Phase-contrast images are shown in the top-left corner of each fluorescence image. Loss of green fluorescence (mNeonGreen-LexA) indicates the presence of DNA damage and induction of the SOS response. Yellow stars indicate cells devoid of fluorescence. Red outlines indicate cell boundaries detected from phase-contrast images. Scale bar in (C-D) indicates 2 µm. Data was processed from two individual biological repeats where 100 cells were detected and analysed. Wild-type corresponds to a strain encoding the ectopic *dnaD-ssrA* cassette that was depleted from IPTG and relied on the endogenous copy of wild-type *dnaD* (WT) to sustain growth (CW1148); 7A (CW1149).

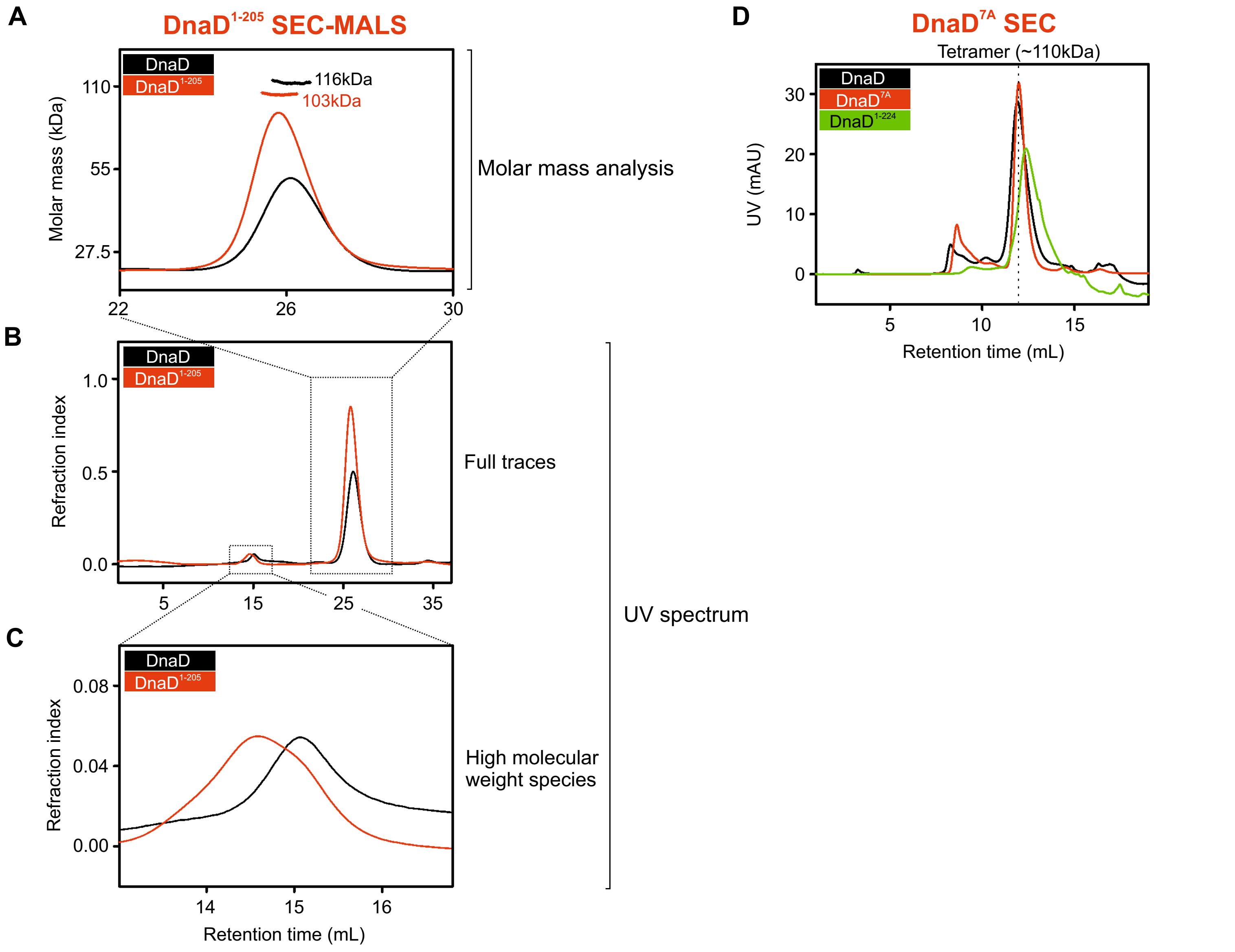

Figure S4. Size analyses of DnaD variants. (A-C) SEC-MALS analysis of purified DnaD protein variants. The wild-type protein (DnaD, black) or a truncation of the DnaD^{CTT} (DnaD$^{1\text{-}205}$, red) were both resolved as tetrameric species. The UV spectrum (continuous lines) was normalised as a relative refraction index. **(A)** The molar mass corresponding to each protein is shown as shorter/thicker lines overlapping the different peaks of the refraction index. Masses corresponding to each peak are annotated on the plot. **(B-C)** Full trace of the UV-spectrum represented as a refraction index during the SEC experiments showing that the DnaD variants did not display major fractions of aggregates (high molecular weight species). **(D)** SEC analyses of DnaD wild-type and DnaD^{7A} and DnaD$^{1\text{-}224}$ show that the 7A allele and C-terminal truncation had a similar UV-spectrum to the wild-type protein. Both proteins were able to form tetrameric species (dotted vertical line).

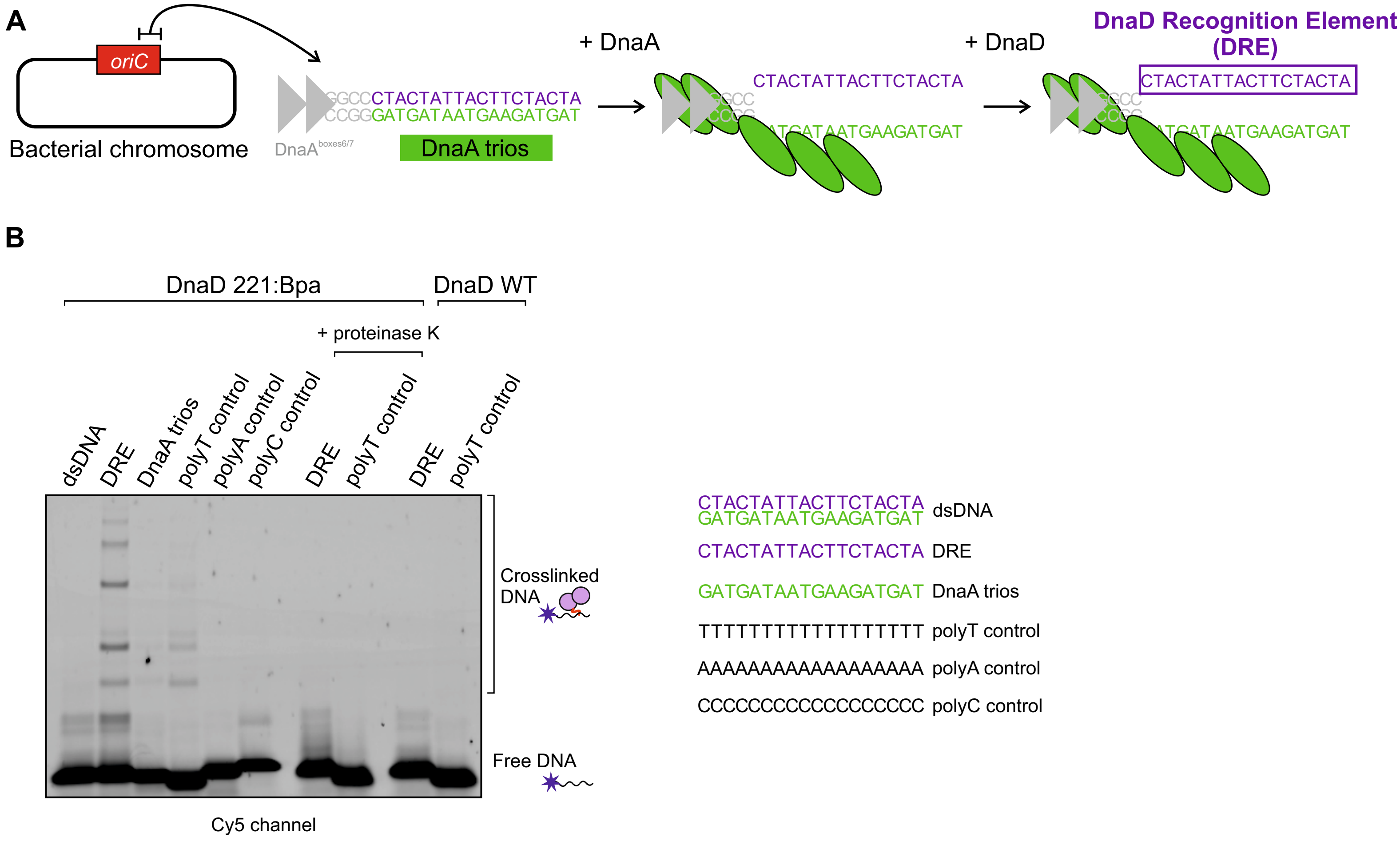

Figure S5. Crosslinking of DnaD to ssDNA substrates. (A) Illustration of the proposed basal origin unwinding mechanism in *B. subtilis*. The closed origin region *incC* is bound by DnaA on DnaA boxes, which leads to partial melting of the origin and strand separation via DnaA oligomer formation on the DnaA-trios. The complementary sequence to the DnaA-trios is proposed to be a specific binding substrate for DnaD. **(B)** Bpa crosslinking assay showing that DnaD interacts with the DRE via the amino acid residue 221 in the DnaD^{CTT}. Incubation with Cy-5 labelled oligonucleotides shows that DnaD 221:Bpa does not crosslink with a dsDNA complex formed by the DRE and DnaA-trios. When used as ssDNA substrates, DnaD 221:Bpa specifically crosslinked the DRE. Treatment via proteinase K shows that binding of DnaD 221:Bpa to ssDNA was specific. Incubation with wild-type DnaD shows that the native protein was not able to be crosslinked onto ssDNA. Oligonucleotide sequences are indicated to the right of the gel: dsDNA (oSP1132:oCW1040), DRE (oSP1132), DnaA trios (oSP1133), polyT control (oSP1135), polyA control (oSP1137), polyC control (oSP1134).

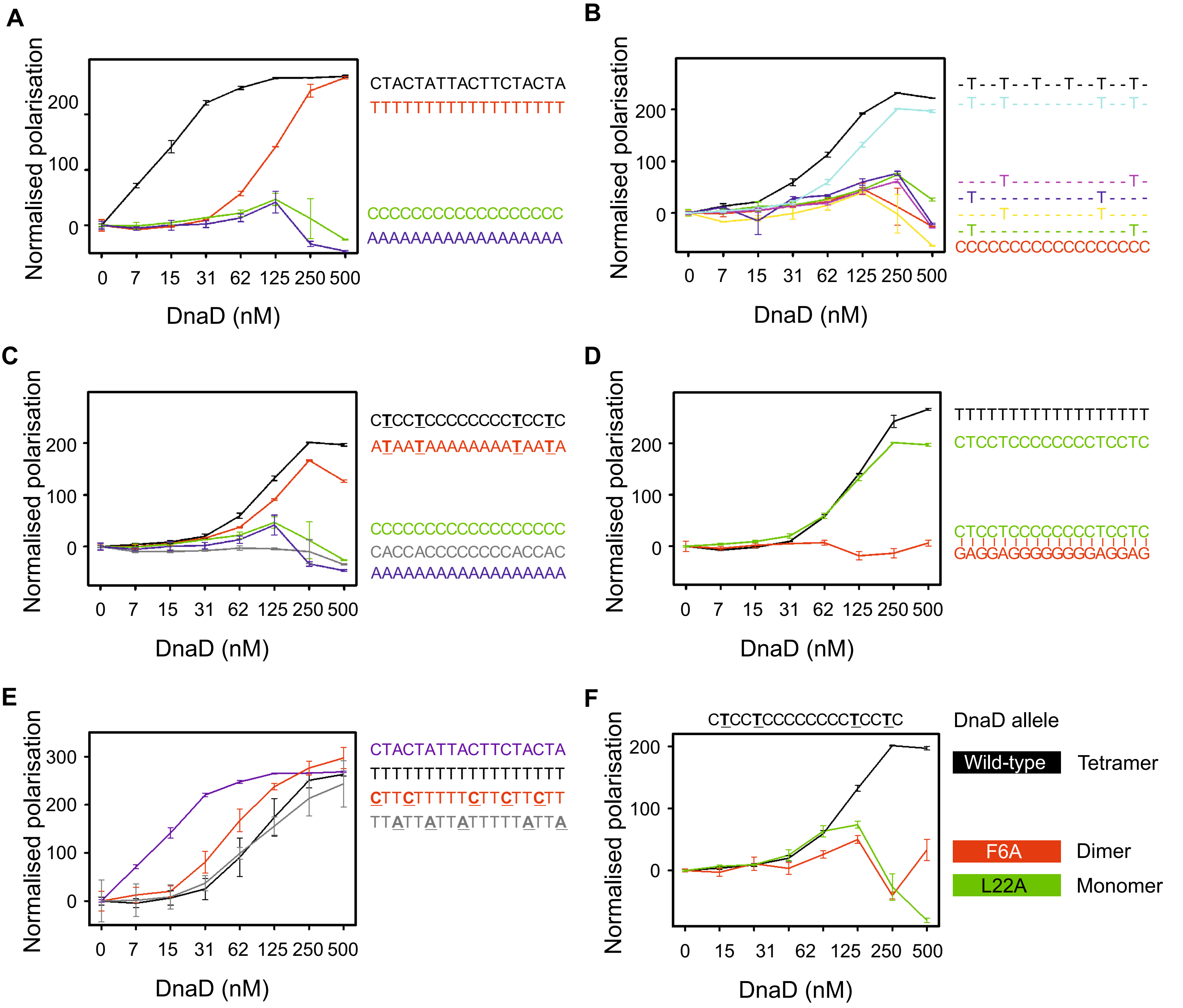

Figure S6. Two 5'-TnnT-3' repeats are required for DnaD ssDNA binding *in vitro*. (A-F) show fluorescence polarisation analyses of DnaD binding to a range of DNA substrates. **(A)** shows wild-type DnaD binding to homopolymers of size and sequence comparable to the DRE. The black line shows binding to a sequence corresponding to the DRE (oCW1039), the red line to a polyT18 ssDNA (oCW1088), the green line to a polyC18 substrate (oCW1089) and the blue line to a polyA18 (oCW1090). **(B)** shows wild-type DnaD binding to 5'-TnnT-3' motifs located within an inert ssDNA substrate and that all thymidines equally contribute to ssDNA binding. Black (oCW1139), cyan (oCW1128), pink (oCW1167), blue (oCW1166), yellow (oCW1155), green (oCW1127) and red (oCW1089). **(C)** shows that wild-type DnaD binding is specific to 5'-TnnT-3' motifs located within otherwise inert ssDNA substrates, and that dual repeats of 5'-AnnA-3' motifs are not recognised. Black (oCW1128), red (oCW1129), green (oCW1089), grey (oCW1216) and blue (oCW1090). **(D)** shows that wild-type DnaD only binds a 5'-TnnT-3' dual repeat in the context of ssDNA. Black polyT18 control (oCW1088), green 5'-TnnT-3' dual repeat as ssDNA (oCW1128), red 5'-TnnT-3' dual repeat as dsDNA (oCW1128:oCW1527). **(E)** shows that re-introducing cytosines at physiological positions matching those of the DRE in a readily bound polyT18 subtrate leads to a mild increase in wild-type DnaD binding specificity, whereas re-introducing adenines produced a binding profile similar to that of DnaD to a polyT18 ssDNA. Purple (oCW1039), black (oCW1088), red (oCW1054), grey (oCW1691). **(F)** shows that oligomerisation mutants of DnaD (F6A and L22A alleles) are unable to bind dual 5'-TnnT-3' motifs located within an otherwise inert polyC18 substrate (oCW1128) compared to wild-type DnaD. Black (wild-type DnaD), red (dimer DnaD^{F6A}) and green (monomer DnaD^{L22A}). Error bars in (A-F) show the standard error of the mean for 2-5 biological replicates.

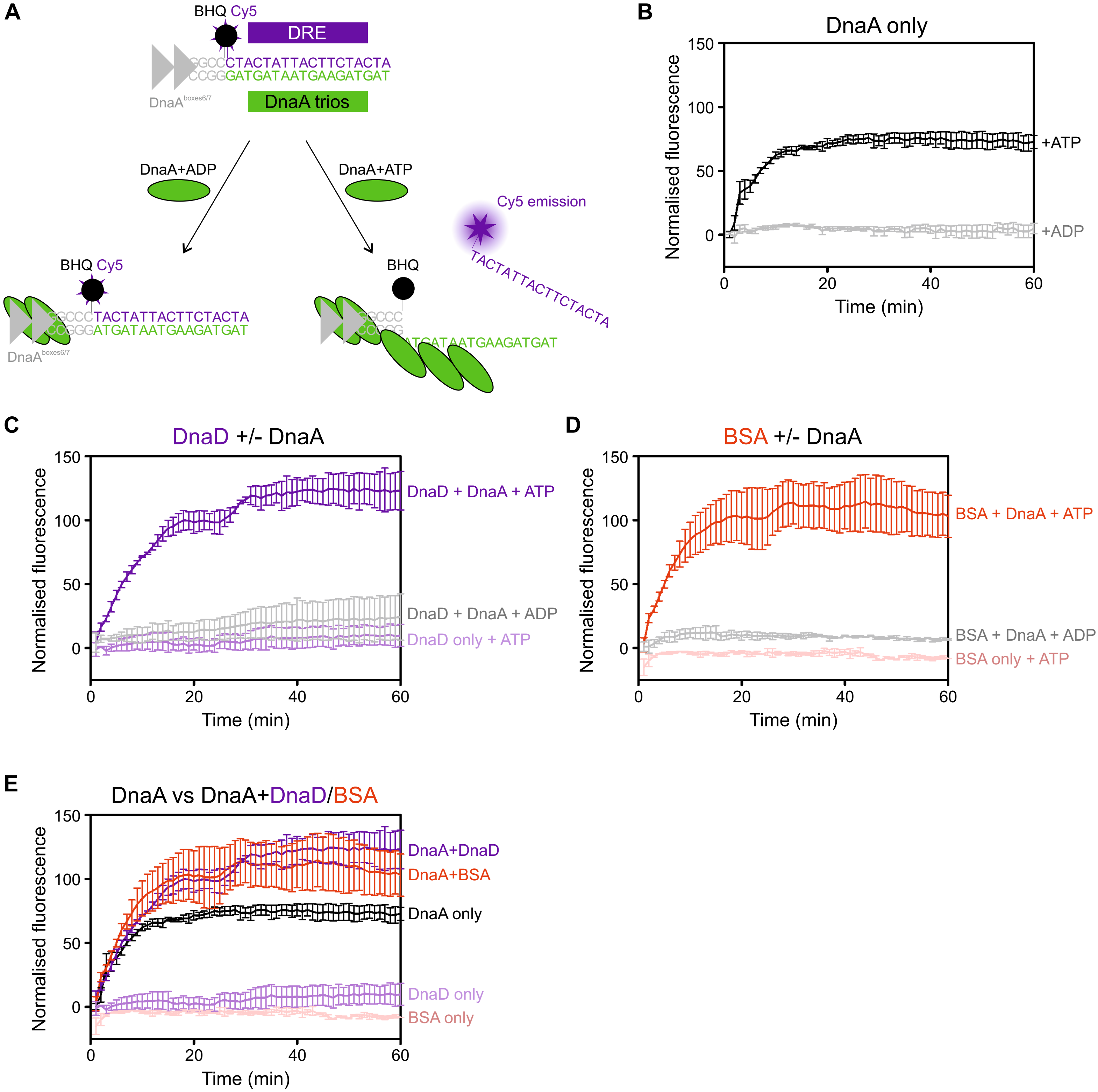

Figure S7. DnaD does not specifically contribute to substrate unwinding via DnaA. **(A)** Illustration of the strand separation assay setup used to detect DnaA-directed unwinding of DNA substrates. Three oligonucleotides are annealed to mimick the *B. subtilis* origin unwinding region including DnaA boxes (dsDNA binding via DnaA) and the DnaA trios/DRE region. The bottom strand is continuous and unlabelled. The top strand corresponding to the DnaA boxes and GC-rich region is labelled with a black-hole quencher (BHQ) at the 3'-end and the DRE is labelled with Cy5 at the 5'-end. As a fully dsDNA probe, the BHQ quenches fluorescence emitted by the Cy5 group. Upon incubation with DnaA and ADP, DnaA binds DnaA boxes, cannot engage the DnaA trios and no fluorescence remains quenched. In the presence of ATP, DnaA binds DnaA boxes and forms an oligomer on the DnaA trios, thereby displacing the DRE probe and allowing emission of Cy5 fluorescence. **(B-E)** Strand separation assays performed with the same probe in the presence of various proteins. DNA substrate: oHM558/oHM778:oHM590. Background corresponding to the basal fluorescence of the DNA probe was subtracted from the curves. Error bars show the standard error of the mean for three biological replicates. **(B)** shows that DnaA only separates strands in the presence of ATP. **(C)** shows that DnaD does not separate strands on its own in the presence of ATP, and that DnaA can still unwind substrates when incubated with DnaD and ATP. **(D)** shows that BSA (control protein) does not separate strands on its own in the presence of ATP, and that DnaA can still unwind substrates when incubated with BSA and ATP. **(E)** shows that strand separation is comparable when using DnaD or BSA along DnaA in the presence of ATP. DnaD or BSA cannot separate strands without DnaA.

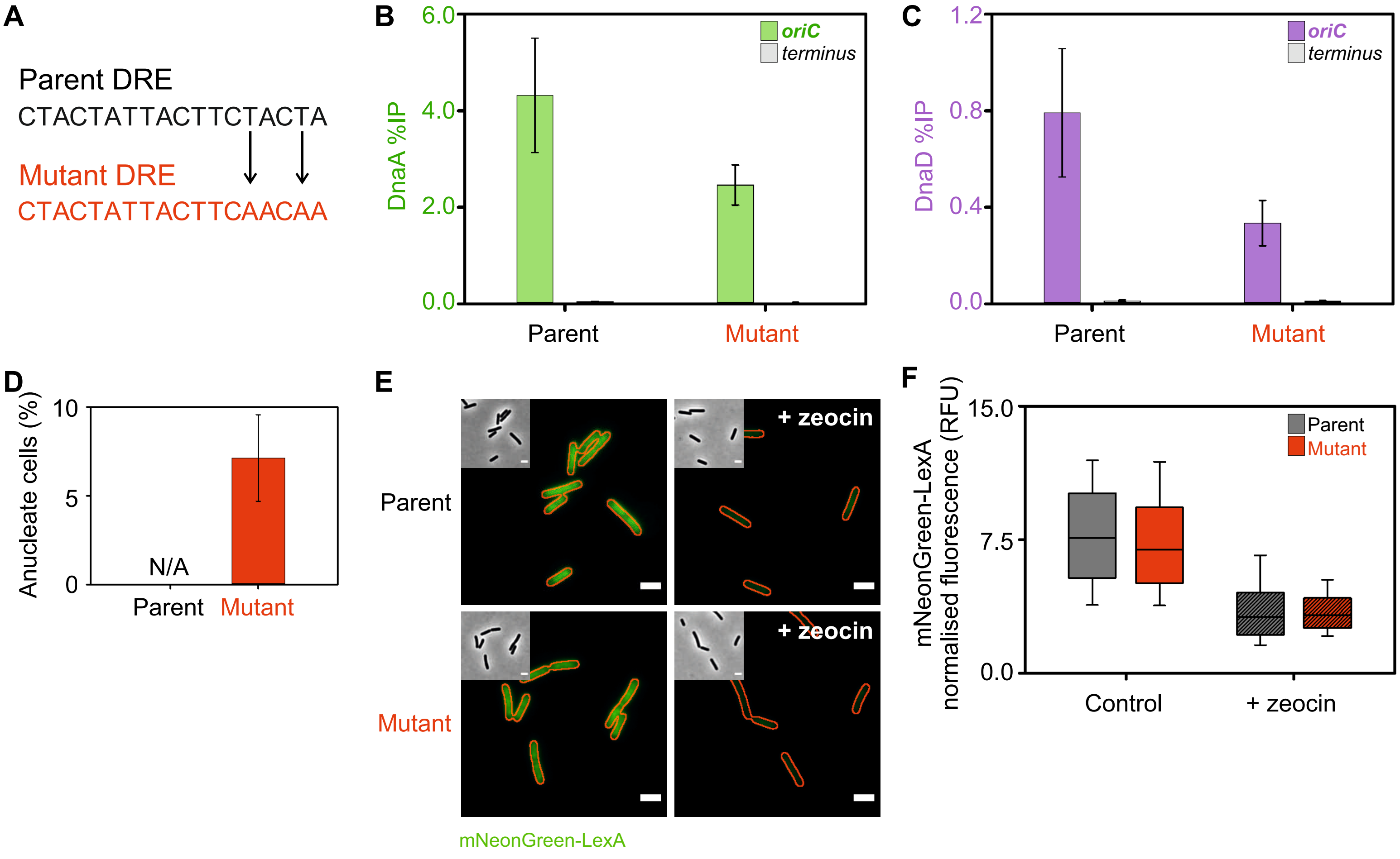

Figure S8. *In vivo* analysis of origin variants. (A) shows the mutation that was introduced in the DRE *in vivo.* The last 5'-TnnT-3' motif present in the parent sequence was mutated to 5'-AnnA-3'. **(B-C)** show the %IP detected for the recruitment of DnaA and DnaD to the origin of replication variants using ChIP. Parent indicates a wild-type strain (168CA) and mutant a strain harbouring the mutation depicted in panel (A) (CW691). **(B)** shows recruitment of DnaA and **(C)** shows recruitment of DnaD. Primers used to amplify the origin in panels (B-C) annealed within the *incC* region. Error bars in panels (B-C) show the standard error of the mean for three biological replicates. **(D)** shows that the origin mutant displayed in panel (A) produces anucleate cells. Quantification was performed from two biological repeats, where over 750 cells were counted for each strain. N/A indicates that no anucleate cells were found and the error bar indicates the standard error of the mean. **(E)** Representative fluorescence microscopy images of origin variants showing that the DRE mutation shown in (A) does not lead to DNA damage. Phase-contrast images are shown in the top-left corner of every image. Loss of green fluorescence (mNeonGreen-LexA) indicates the presence of DNA damage and induction of the SOS response. Red outlines indicate cell boundaries detected from phase-contrast images. Zeocin was used as a positive control for DNA damage. Scale bar indicates 2 µm. Parent corresponds to a wild-type strain encoding the endogenous *mNeonGreen-lexA* cassette (CW1138) and mutant indicates the origin variant shown in (A) (CW1139). **(F)** Single-cell analysis performed on the data obtained from experiments shown in (E) indicates that intracellular levels of mNeonGreen-LexA are comparable between wild-type and mutant origins. Normalised intracellular fluorescence indicates the average fluorescence intensity per cell area. Zeocin was used as a positive control. Image analysis was performed from two individual biological replicates where over 100 cells were detected and analysed per experimental condition.

A

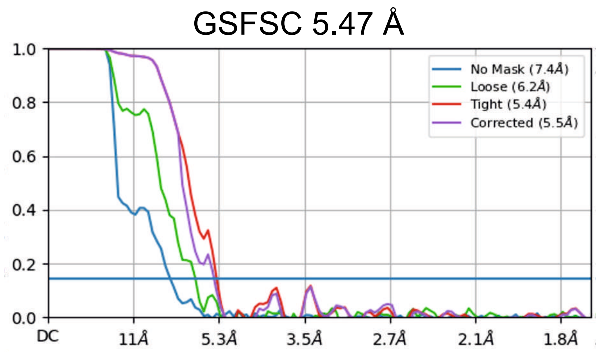

B

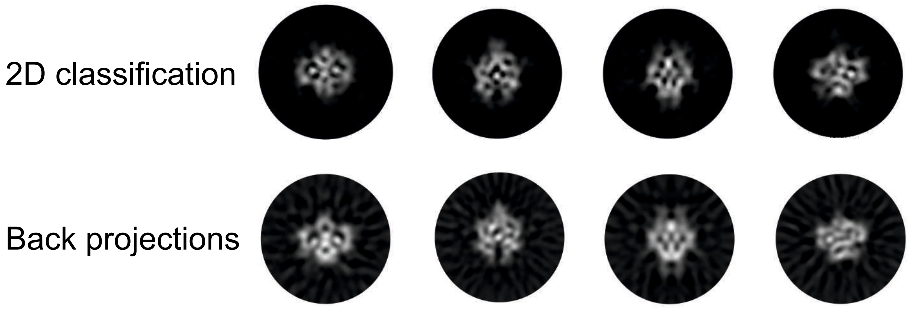

Figure S9. DnaD structure solved by cryo-EM. (A) Overall resolution of the DnaD^{NTD} tetramer derived from 443,529 particles using the FSC=0.143 criteria. **(B)** Back-projections of the cryo-EM structure onto 2D classes.

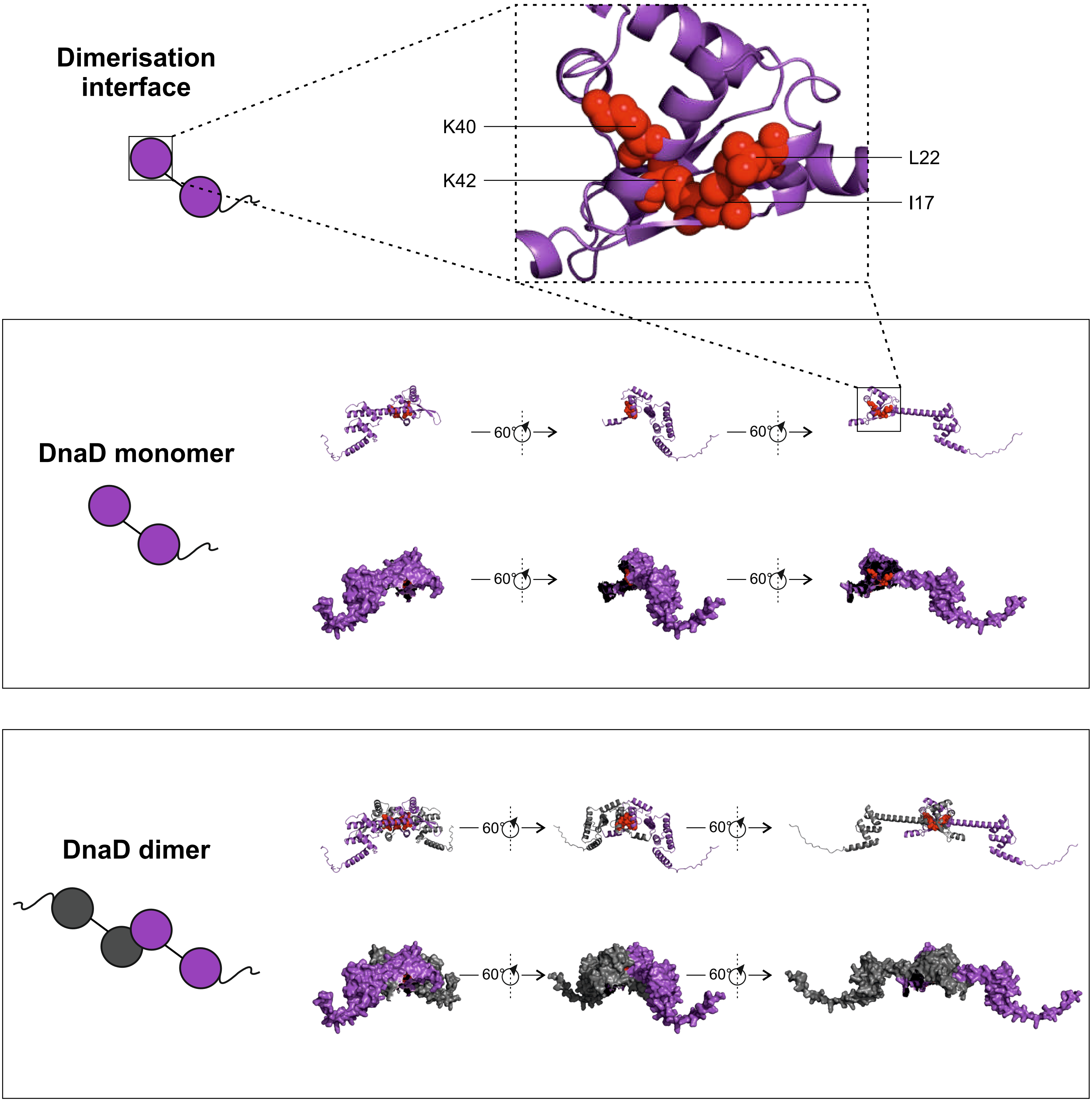

Figure S10. DnaD dimer formation model. Model of DnaD oligomerisation pathway for monomer to dimer transition based on the available DnaD structures (N-terminal domains from PDB 2V79 and C-terminal domains from 2ZC2) with amino acid residues involved in dimerisation shown in red.

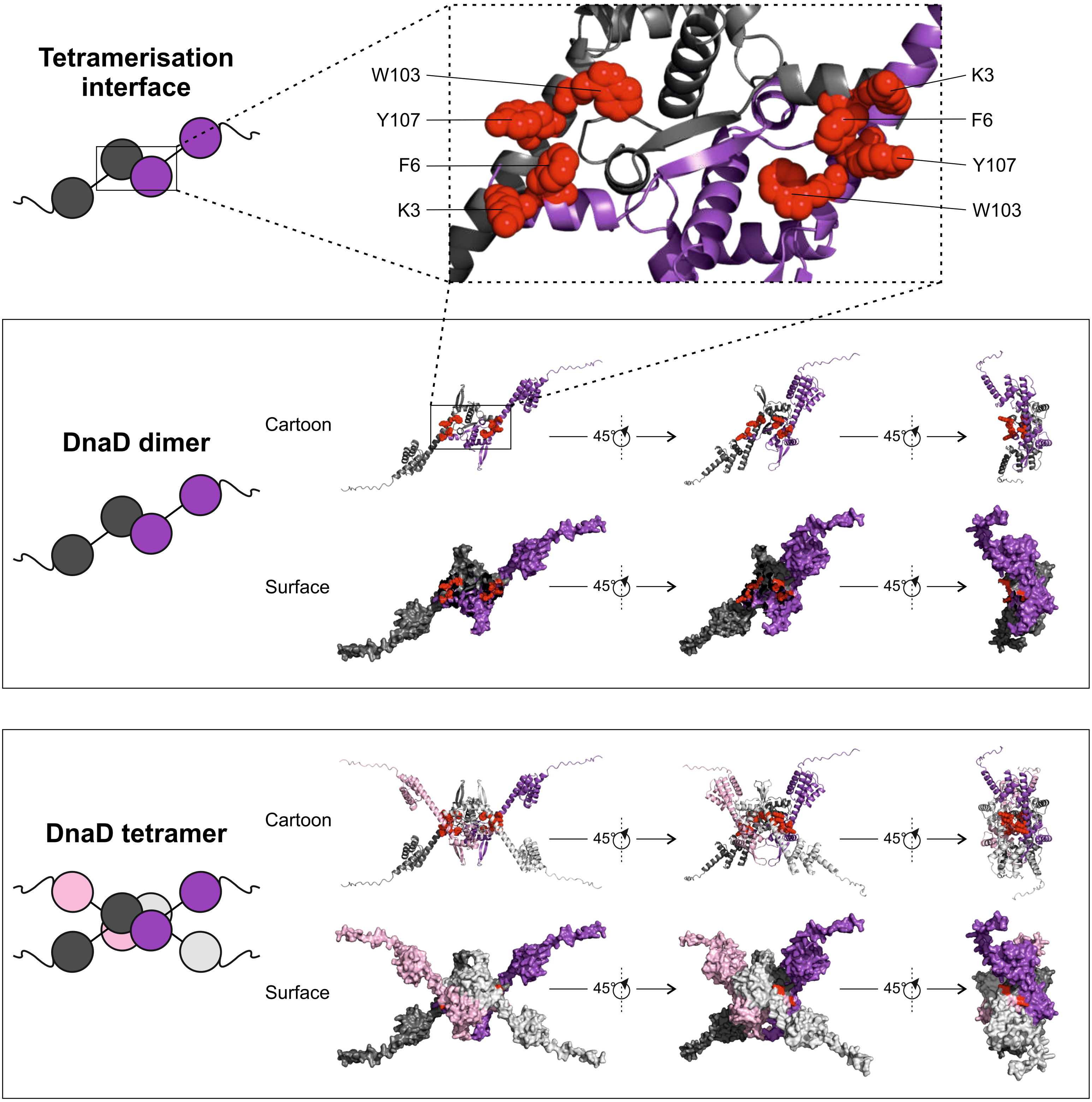

Figure S11. DnaD tetramer formation model. Model of DnaD oligomerisation pathway for dimer to tetramer transition based on the available DnaD structures (N-terminal domains from PDB 2V79 and C-terminal domains from 2ZC2) with amino acid residues involved in tetramerisation shown in red.

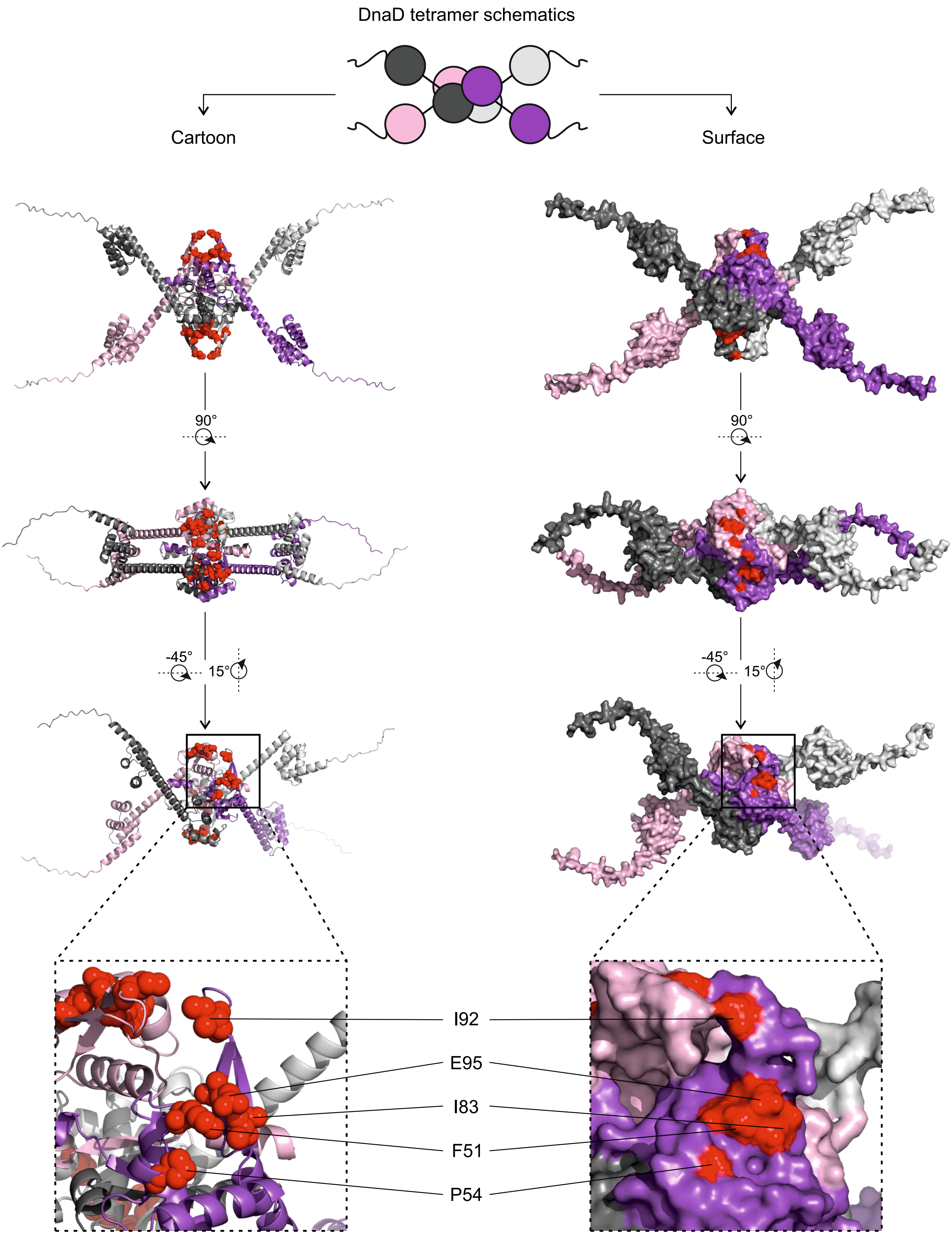

Figure S12. DnaD interface involved in binding DnaA domain I. DnaD tetramer model based on the available DnaD structures (N-terminal domains from PDB 2V79 and C-terminal domains from 2ZC2) with amino acid residues involved in binding DnaA shown in red.

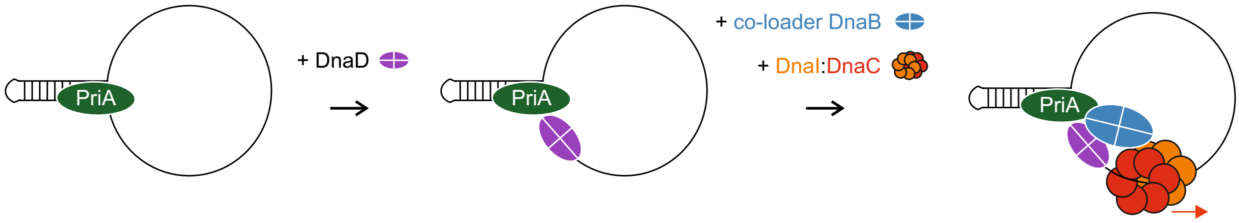

Figure S13. Model for helicase recruitment and loading in *B. subtilis* during PriA-dependent replication restart at a single-strand origin (*sso*).
